# Model-based control for exoskeletons with series elastic actuators evaluated on sit-to-stand movements

**DOI:** 10.1186/s12984-019-0526-8

**Published:** 2019-06-03

**Authors:** Jonas Vantilt, Kevin Tanghe, Maarten Afschrift, Amber K.B.D Bruijnes, Karen Junius, Joost Geeroms, Erwin Aertbeliën, Friedl De Groote, Dirk Lefeber, Ilse Jonkers, Joris De Schutter

**Affiliations:** 1 the Robotics Research Group, the Department of Mechanical Engineering, KU Leuven, Celestijnenlaan 300, Leuven, Belgium; 2 Department of Kinesiology, KU Leuven, Tervuursevest 101, Leuven, Belgium; 3 Robotics and Multibody Mechanics Research Group, Department of Mechanical Engineering, VUB, Pleinlaan 2, Brussels, Belgium; 4 Flanders Make, Lommel 3920, Belgium

**Keywords:** Model-based control, Exoskeleton, Sit-to-stand, Assistive robots, Series elastic actuator, Paraplegia, Muscle weakness

## Abstract

**Background:**

Currently, control of exoskeletons in rehabilitation focuses on imposing desired trajectories to promote relearning of motions. Furthermore, assistance is often provided by imposing these desired trajectories using impedance controllers. However, lower-limb exoskeletons are also a promising solution for mobility problems of individuals in daily life. To develop an assistive exoskeleton which allows the user to be autonomous, i.e. in control of his motions, remains a challenge. This paper presents a model-based control method to tackle this challenge.

**Methods:**

The model-based control method utilizes a dynamic model of the exoskeleton to compensate for its own dynamics. After this compensation of the exoskeleton dynamics, the exoskeleton can provide a desired assistance to the user. While dynamic models of exoskeletons used in the literature focus on gravity compensation only, the need for modelling and monitoring of the ground contact impedes their widespread use. The control strategy proposed here relies on modelling of the full exoskeleton dynamics and of the contacts with the environment. A modelling strategy and general control scheme are introduced.

**Results:**

Validation of the control method on 15 non-disabled adults performing sit-to-stand motions shows that muscle effort and joint torques are similar in the conditions with dynamically compensated exoskeleton and without exoskeleton. The condition with exoskeleton in which the compensating controller was not active showed a significant increase in human joint torques and muscle effort at the knee and hip. Motor saturation occurred during the assisted condition, which limited the assistance the exoskeleton could deliver.

**Conclusions:**

This work presents the modelling steps and controller design to compensate the exoskeleton dynamics. The validation seems to indicate that the presented model-based controller is able to compensate the exoskeleton.

## Background

Performing activities of daily living (ADL) is a challenge for people affected by muscle weakness or neurologic disorders, like paraplegia. Crutches and wheelchairs provide help to perform ADL. However, these devices do not encourage the normal use of muscles and they lead to disuse and further deterioration of lower-limb muscle function for ADL. In contrast, assistive lower-limb robots have the potential to support ADL and to encourage active participation of the user, making them valuable additions to or replacements of wheelchairs and crutches. To narrow the focus, this paper looks at assistive devices that are not stationary, i.e. fixed to the ground, and therefore allow overground walking. To achieve this, these devices need to be compact, lightweight and need to have energy autonomy. These requirements have a large impact on both design and control decisions. Assistive robots allowing overground walking, worn on the lower limbs and covering all joints are referred to as exoskeletons in this paper. The term actuated orthoses on the other hand refers to assistive robots that span only one or two joints in this paper.

Young and Ferris [1] point out the variety in control methods and the absence of convergence towards one or a class of controller methods. The variety in control strategies in assistive robotics is further backed up by the review work of Yan et al. [2]. They classify six groups of assistive controllers of which predefined trajectory and model-based control strategies are the most common. Predefined trajectory controllers are often used for gait trainers and exoskeletons for complete paraplegic persons. These predefined trajectories are enforced with position or impedance control [3, 4]. For example, devices for spinal cord injuries move the user’s limbs according to these predefined trajectories. In rehabilitation robotics, the enforced trajectories train muscle strength and coordination [5–8]. Here, the impedance controller is widely used: the more the subject deviates from the desired trajectory, the more assistance the rehabilitation device delivers to guide the user back to the desired trajectory, which increases user involvement during training [9, 10]. In contrast, enforcing a trajectory with an impedance controller is undesirable in an exoskeleton supporting voluntary capabilities.

Our work focusses on exoskeletons that assist the voluntary capabilities of people suffering from mild forms of paraplegia or muscle weakness. Enforcing a trajectory with an impedance controller is undesirable in such exoskeletons [1], because these exoskeletons need to keep the user in charge of the motion. Model-based control strategies, as described by Yan et al. [2], are more appropriate for exoskeletons assisting the voluntary capabilities of the user. These methods rely on human-exoskeleton models and can often compensate gravity, determine the assistance and even incorporate balance criteria. Also controllers utilizing the muscle activity [11, 12] are classified as model-based controller in the work of Yan et al. [2]. In this paper, model-based control relies on physical models of the dynamics and contacts of the system (exoskeleton and human) to determine the actuation from the motors. In many cases, such controllers only use exoskeleton gravity compensation [8, 10], even though the influence of the dynamics cannot be ignored [13, 14]. Only a few controllers compensate the complete exoskeleton dynamics [14, 15], as this requires a full dynamic model of the exoskeleton. The output of these model-based controllers are the torque needed at each joint.

The widely used Series Elastic Actuators (SEA) [6, 7, 13] facilitates torque control in exoskeletons. This type of actuator has a spring(like) element in series with the actuator. By measuring the extension and impression of the springs, the SEAs transform the torque/force control problem to a position control problem using their compliance. This facilitates a torque-based control strategy [16–18]. In addition, these actuators increase user safety and comfort, which is important in applications directly involving human interaction. The spring acts as a buffer for impact and reduces the actuator inertia felt by the user. An added advantage is that the springs are able to store energy. Although SEAs are unable to lock this stored energy, they do allow to reduce peak motor power when designed properly [19]. These advantages of SEAs come at a cost in the form of a reduced positioning bandwidth and an increased number of mechanical parts, which may increase the overall weight.

When exoskeletons or orthoses already have low mass and inertia by design, compensating them is less crucial to achieve beneficial results. Only a few ankle orthoses have shown to be effective, as evidenced by a reduced metabolic cost for walking or running [20–22]. All these orthoses have low mass and rotational inertia, either by putting the motors on a separate control station [20, 21] or because they have no motors at all [22]. However, for complete exoskeletons, which need to carry their own batteries and actuators, the mass and rotational inertia is much higher, requiring a substantial part of the actuators capabilities. If the exoskeleton dynamics are not compensated for by the controller, the effective assistance provided to the user is reduced.

In contrast to the different control strategies in assistive robotics [2], in humanoid robotics, a model-based control strategy that accounts for the full robot dynamics is common practice [23–25]. Additionally, these models can also estimate ground reaction forces [26, 27], needed as input to the dynamics models. In order to achieve model-based control, humanoid robots are almost always torque-controlled, often using force cells. The fundamental difference between humanoid robots and exoskeletons is the presence of the human, which greatly impacts design and control. The weight and size limits for exoskeletons result in actuators which operate close to their limits. On top of that, the human contributes to the actuation, possibly affecting stability. Furthermore, the exoskeleton needs to adapt itself to the motions of the human operator and their execution speed.

The similarity between humanoid robots and exoskeletons provides a motivation to also move towards model-based control in exoskeletons. The work of Yan et al. [2] contains different types of models that are used in a variety of ways. This makes a comparison between controllers difficult [1]. Irrespective of the used assistive control strategy [2], the device attached to the person also consumes part of the actuation. If this is not compensated for, the provided assistance is affected. A model-based control strategy can compensate these actuation needed by the exoskeleton dynamics. In this way, the exoskeleton follows the motions of the human such that the human does not feel the extra weight and inertia of the exoskeleton. Compensating the exoskeleton in a model-based way allows us to more properly compare different assistance strategies for exoskeletons.

A key element in the model-based exoskeleton dynamics compensation is the contact modelling with the environment. A change in contact situation leads to a change in the compensation controller. In the literature, Finite State Machines (FSM) are often used to specify different (discrete) states of the controller [1]. Depending on the phase of the motion, different states are defined which can have their own control strategy. In this way, position and force control can be interchanged depending on the phase of motion [6, 14]. Many of the states of the FSMs implicitly relate to the contact with the environment. For example, the swing and stance phases of gait indicate if the foot makes contact with the ground. In this work, the states of the FSM are directly linked to the contact situations with the environment.

The computation of the assistive torques can also rely on a model-based strategy. Such a model-based approach relies on a musculoskeletal model of the user, comprising two parts. First, the skeletal, dynamics model calculates the torques required at each joint for a given task. Second, the muscle model estimates the torque which the user is able to deliver with his muscles. The work of Carmichael et al. [28] shows a method to estimate the assistance needs of a person with respect to the task at hand relying on muscle models. Hence, human capability is expressed at muscle level and accounts for the changing lever arms of the muscles, the muscle force-length and force-velocity properties, and the effect of bi-articular muscles. This makes the model usable for any activity at any speed. How the muscle strength needs to be identified is not tackled, nor is the influence of muscle fatigue accounted for. A model that is less computationally intensive expresses human capability at the joint level by imposing joint torque limits. However, this last model neglects the mentioned muscle effects. Hence, these joint torque limits are only valid for a specific motion at a specific speed and hence are not generalizable.

In order to achieve both the compensation of the exoskeleton dynamics and the user-specific assistance, this paper presents a novel model-based torque control approach for exoskeletons. The focus lies on the compensation of the exoskeleton dynamics, the model-based assistance is computed off-line and the assistance is not the focus of this paper. Both the model-based exoskeleton dynamics compensation and the model-based human assistance are generalizable to other motions, because the models are usable in any contact situation. The first contribution is the extensive modelling of the exoskeleton and its interactions with the environment. The boundaries of the models and assumptions made during modelling are presented. The modelling starts from a model-based control scheme and modelling procedure. The second contribution is the design of the feedforward control signal for the SEA. This feedforward signal is important to obtain good torque tracking, since the bandwidth of the feedback position control is limited. The third contribution is the validation of the control method on an exoskeleton for sit-to-stand motions. A sit-to-stand motion is chosen as validation because this motion is less reported on in the literature and it is not a cyclic motion such as overground walking. The absence of the cyclic characteristic makes forces and torques harder to predict.

The Methods section presents the model-based control approach for assistive robots. The general control scheme, modelling of exoskeleton and environment and the feedforward for SEAs are presented. The Sit-to-stand Exoskeleton section explains the implementation of the model-based control methods on an exoskeleton for standing up and sitting down. The used exoskeleton hardware and sensor are also presented. The experiments and results are described in the next section, followed by the discussion and conclusion.

## Methods: Model-based Control

Because all controllers do depend on hardware to some extent, modelling procedures and assumptions behind the control design are of great importance for other researchers working on different hardware. However, for many existing exoskeletons, a detailed description of the control strategy is not available, as pointed out in [1]. To encourage the use of and reporting of dynamic models of assistive devices in control, a systematic modelling procedure is presented in Table 1, focusing on a single activity (like walking). The contact with the environment plays a key role in the modelling procedure. Although the modelling procedure presented in Table 1 is obvious, the importance of the contact situations of the exoskeleton cannot be emphasized enough. The different contact situations directly influence controllers.

**Table 1:** Modelling procedure for an activity on an existing exoskeleton (hardware is given)

step no.	name	description
1	list sensors	list all sensor information available for the exoskeleton controller
2	contact	determine and characterize the different environment contact situations in the activity
3	detect	decide how to detect the transition in environment contact using the available sensor data
4	model	model the dynamic models of each different environment contact situation and their transitions

**Fig. 1:**
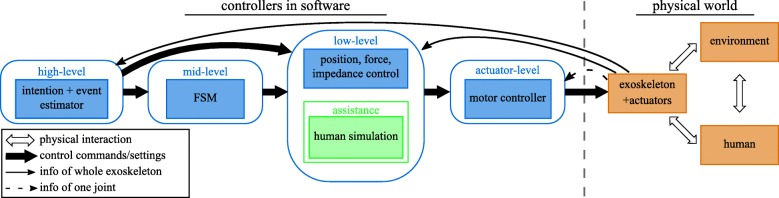
General control scheme for an exoskeleton. The physical system in red is separated from the control by the dashed line. The control is divided into four hierarchical levels, with a few keywords to indicate their function. The thin lines represent data flow from the available exoskeleton sensors. The thick lines represent the control/decision commands to a lower control level. The assistance block in green transforms the sensor data to desired assistance in the low-level control block

This modelling procedure is supplemented by a general control scheme in Fig. 1. This control scheme differs from other control schemes, like the one of Tucker et al. [29], by having a strong link with the modelling procedure. Our control scheme has four control levels: actuator, low-level, mid-level, and high-level control as indicated in the figure. Their relation with the modelling steps is explained as follows. The actuator control level uses the model of one actuator, the low-level controller uses the model of the whole exoskeleton and the mid- and high-level controller relate to modelling the ground contact of the exoskeleton.

The lowest control level is the *actuator level*, which sends commands to a single actuator unit, consisting of a motor and possibly a spring. Our focus is on electric motors. Typical control approaches use velocity controllers or torque controllers. The choice between a velocity or torque controller influences how the dynamic models are used in the low-level control, as the control commands and feedforward commands differ. The inputs of the actuator controller are the sensor data of the actuated joint and the desired reference control signal.

The *low-level* controller sends reference commands to all actuator controllers. The control strategy is either force, impedance or position control. This control level utilizes dynamic models of the exoskeleton and contact models with the environment. The high complexity and the lack of information make it impractical to model every part of the physical world in Fig. 1. However, it is usually feasible to model the exoskeleton and its changing interactions with the environment, which is the result of modelling step 4. Inputs of this control level are control gains and modelling parameters supplied by the mid-level controller, together with sensor data of the whole exoskeleton.

The assistance depends on the control strategy and, consequently, is part of the low-level controller. In impedance control, the assistance is directly related to the chosen impedance and the error with respect to the imposed reference trajectory. In force control, the assistance is a force or torque which the assistive robot provides to the human. In this work, the assistance torque complies with the assistance-as-needed paradigm. Relying on the musculoskeletal model of the human and the sensor data of the exoskeleton, the capability gap [28, 30] is calculated. This capability gap is the difference between the torque demanded by the motion of the human and the torque available by the human muscles.

The *mid-level* controller decides which model the low-level controller should use, depending on the contact. To achieve this, the mid-level controller changes control gains, models and/or model parameters of the low-level controller. In addition, the mid-level controller also triggers when to start and stop giving assistance when this assistance is not always active such as in the capability gap calculated assistance. The mid-level controller is an FSM, a discrete controller. The states are related to the different contact situations of the activity, which is an outcome of modelling step 2 in Table 1. The inputs of this control level are the events (important occurrences) detected by the high-level controller.

While the mid-level controller implements how the contact models should change, the *high-level* controller implements *when* these changes need to occur. The high-level controller does this by sending events to switch the state of the FSM in the mid-level controller. The events are related to the current and future contact situation with the environment. Hence, the high-level controller consists of several intent and event estimators, which are the outcome of modelling step 3. These estimators detect or predict an upcoming change in contact using all the available sensor data. The predictions are needed to ensure a continuous transition in the models used in the low-level controller.

The following subsections present the modelling steps of the exoskeleton and the contact situations and how to compensate the exoskeleton dynamics in the controller. In the first subsection, the exoskeleton dynamics model is presented and the compensation of the exoskeleton dynamics is derived. In the second subsection, the impact of the contact on the dynamics is shown and the contact modelling step is explained. The third subsection tackles the feedforward control command for the actuator control level to improve the performance of the controller.

### Modelling and Compensation of the Exoskeleton Dynamics

The controller needs to actively compensate the dynamics of the exoskeleton, as these dynamics have a negative effect on the performance. Any exoskeleton which physically interacts with a person unavoidably adds mass and rotational inertia to the person. When the controller compensates for these added dynamics, the exoskeleton delivers the torques to move itself. As the person observes a reduced exoskeleton mass and rotational inertia, this control mode is referred to as transparent control. The exoskeleton dynamics compensation does not limit itself to gravitational effects [8, 10, 12, 31], but encompasses the whole dynamics including inertial effects, which are not negligible [13, 14].

**Fig. 2:**
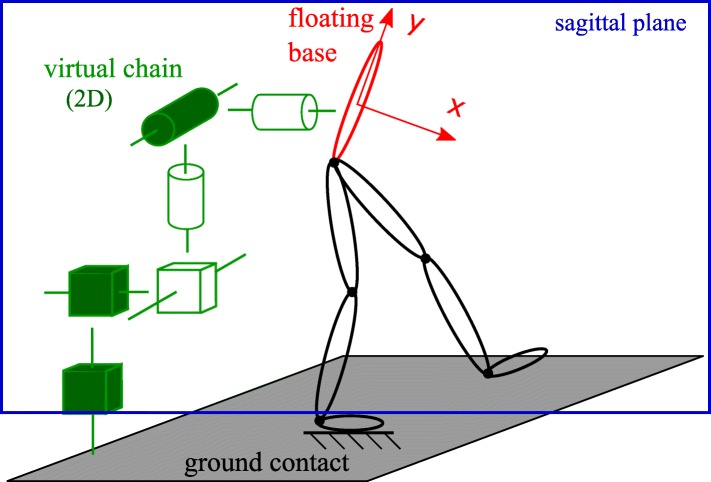
Floating base model of the exoskeleton. The floating base representation of an exoskeleton is characterized by a virtual, unactuated chain. This chain consists of six dofs: three translations and three rotations in 3D. When using a 2D model this reduces to two translations and one rotation, shown by the filled dofs. These actuators lie in one plane, the sagittal plane, shown in blue

A floating base dynamic model [32], see Fig. 2, is chosen to calculate the exoskeleton torques through inverse dynamics. This model is characterized by a virtual kinematic chain that connects the floating base to the ground. This virtual chain represents all degrees of freedom (dof) of the *floating* base, but is not actuated. In order to move the exoskeleton in the world, additional actuated paths towards the ground are needed. Such an actuation path is a chain of exoskeleton links connected to the ground and running up to the base link. Each ground contact applies constraints to the dynamic model and introduces constraint wrenches, consisting of forces and moments. Because most exoskeletons are only actuated in the sagittal plane, a 2D dynamic model suffices to describe them.

**Fig. 3:**
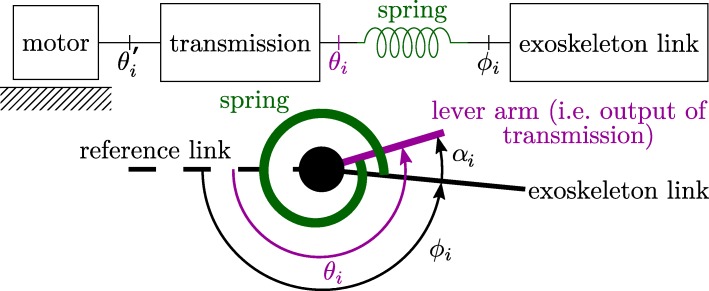
Schematic model of an SEA. An SEA consists of a motor, a transmission, a spring and the outgoing link, as shown by the top schematic. All variables are referenced against the previous link as shown by the dashed line in the lower figure. $\theta _{i}^{*}$ is the motor angle before transmission. *α*_*i*_=*θ*_*i*_−*ϕ*_*i*_ is the spring deflection angle

**Fig. 4:**
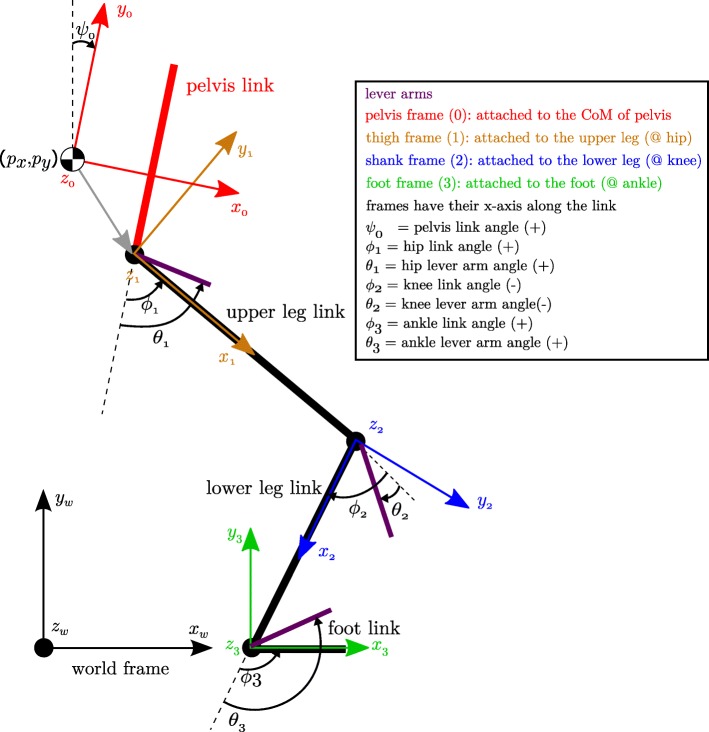
Definition of frames, angles and floating base states variables of the exoskeleton. The thick black lines represent the link of one leg of the exoskeleton viewed from the side (sagittal plane). The joints (hip, knee and ankle are indicated by black circles. The lever arms of the actuated joints are indicated by purple bars. The frame of each link is shown in a different color with all z-axes pointing outward of the paper. The link angles *ϕ*_*i*_ and the lever arm angles *θ*_*i*_ are defined starting from the previous link (=reference link) and according to the right hand rule. The knee joint and lever arm angle are thus negative in this configuration. *p*_*x*_ and *p*_*y*_ represent the position of the center of mass of the floating base link, the pelvis link. The angle *ψ* is the angle of the floating base with respect to the world frame

SEAs are often used in exoskeletons and thus have to be modelled. An SEA consists of a spring in series with the motor as seen in Fig. 3. The motor moves the lever arm with respect to the reference link and a spring between the lever arm and moving link pulls or pushes the moving link. The angles of the lever arm and moving link relative to the reference link are *θ*_*i*_ and *ϕ*_*i*_ respectively. The lever arm angle *θ*_*i*_ is the same as the motor angle after transmission. From here on we will use the term lever arm angle. The angle over which the rotary spring is compressed or extended is *α*_*i*_=*θ*_*i*_−*ϕ*_*i*_. The torque generated by the spring is the actuation torque for driving the moving link, as seen in Eq. (1). SEAs are introduced to the exoskeleton dynamics model by combining a reduced flexible joint dynamic model [33] with a floating base dynamic model [32]:

1 $$ \mathbf{M}(\boldsymbol{q}) \ddot{\boldsymbol{q}} + \mathbf{C}(\boldsymbol{q},\dot{\boldsymbol{q}}) \dot{\boldsymbol{q}} + \mathbf{G}(\boldsymbol{q}) = \mathbf{S}^{T} \boldsymbol{\tau}_{spr} + \mathbf{J}_{c}^{T}(\boldsymbol{q}) \boldsymbol{w}_{c} + \boldsymbol{f}_{h},   $$

2 $$ \mathbf{B} \ddot{\boldsymbol{\theta}} + \boldsymbol{\tau}_{spr} = \boldsymbol{\tau}_{motor},   $$

with the assumption that no energy is dissipated in the contacts (e.g. by sliding friction) and with the following definitions:

- $\boldsymbol {q}, \dot {\boldsymbol {q}}, \ddot {\boldsymbol {q}} \in \mathbb {R}^{r \times 1}$: the state vector, consisting of three floating base variables (*p*_*x*_,*p*_*y*_ and *ψ*) and *n* joint angles *ϕ*_*i*_, i.e. ***q***=[*p*_*x*_ *p*_*y*_ *ψ* *ϕ*_1_ … *ϕ*_*n*_]^*T*^, see Fig. 4 for the definition of all variables,
- $\boldsymbol {\theta }, \dot {\boldsymbol {\theta }}, \ddot {\boldsymbol {\theta }} \in \mathbb {R}^{n \times 1}$: the lever arm state vector,
- $\mathbf {M} \in \mathbb {R}^{r \times r}$: the inertia matrix,
- $\mathbf {C} \in \mathbb {R}^{r \times r}$: the Coriolis and centrifugal matrix,
- $\mathbf {G} \in \mathbb {R}^{r \times 1}$: the gravitational vector,
- $\boldsymbol {\tau }_{spr} \in \mathbb {R}^{n \times 1}$: the spring torques,
- **S**=[**0**_*n*×3_ **I**_*n*×*n*_]: the actuation selection matrix,
- $\boldsymbol {w}_{c} \in \mathbb {R}^{k}$: the vector of all contact forces/moments,
- $\mathbf {J}_{c} \in \mathbb {R}^{k \times r}$: the contact/constraint Jacobian,
- $\boldsymbol {f}_{h} \in \mathbb {R}^{r \times 1}$: the vector of human interaction forces/torques,
- $\mathbf {B} \in \mathbb {R}^{n \times n}$: the diagonal motor inertia matrix after transmission,
- $\boldsymbol {\tau }_{motor} \in \mathbb {R}^{n \times 1}$: the motor joint torques after transmission.

The parameters in **M**,**C**,**G** and **B** consist of the masses, center of masses, rotational inertias and lengths of the exoskeleton links, which are derived from CAD models or are experimentally identified [34, 35]. From here on, the dependence of matrices and vectors on ***q*** or $\boldsymbol {\dot {q}}$ is omitted in the equations. The number of actuated joints of the robot is *n*; *r*=*n*+3 is the number of the floating base model states and *k* is the dimension of the independent contact constraints. For example, with one non-sliding foot flat on the ground *k*=3; with two non-sliding feet flat on the floor *k*=6. When making contact with the ground, the kinematics at the contact points are constrained using the additional equations:

3 $$ \dot{\boldsymbol{p}}_{c} = \mathbf{J}_{c} \dot{\boldsymbol{q}} = 0,   $$

4 $$ \ddot{\boldsymbol{p}}_{c} = \mathbf{J}_{c} \ddot{\boldsymbol{q}} + \dot{\mathbf{J}}_{c} \dot{\boldsymbol{q}} = \mathbf{J}_{c} \ddot{\boldsymbol{q}} + \left(\sum_{j=1}^{r} \frac{\partial \mathbf{J}_{c}}{\partial q_{j}} \dot{q}_{j} \right) \dot{\boldsymbol{q}} = 0,   $$

with ***p***_*c*_=[*x*_*left*_ *y*_*left*_ *Ψ*_*left*_ *x*_*right*_ *y*_*right*_ *Ψ*_*right*_]^*T*^ representing the positions and angle of both feet with respect to the world frame.

The human is modelled as an **independent** disturbance ***f***_*h*_ acting on the exoskeleton at joint level, as seen in Eq. (1). This human input consists of two forces and one moment at the floating base and *n* human joint torques. Another way of modelling the interaction between exoskeleton and human is to identify every interaction point and measure every interaction force and moment. This is not a practical solution, because it requires expensive force cells at every link, potentially multiple ones if single axis force cells are used. These force cells add weight and consume space. The human interactions ***f***_*h*_ represent the assistance delivered by the exoskeleton to the human. To calculate the transparent control torques, the human interactions ***f***_*h*_ are set to zero. When the exoskeleton needs to provide assistance, a desired vector of interaction forces and torques ***f***_*h,des*_ is added to the exoskeleton dynamics compensation torques. From here on, we only consider assistance at the joints, referred to as assistance torques ***τ***_*assist*_. When no interaction forces are desired, the exoskeleton acts transparently.

The spring torques required to compensate the exoskeleton dynamics are derived from the *n* lower rows of Eq. (1):

5 $$\begin{array}{*{20}l} \boldsymbol{\tau}_{dyn} = & \beta_{in} \widehat{\mathbf{M}}_{jo} \ddot{\boldsymbol{q}} + \beta_{in} \widehat{\mathbf{C}}_{jo} \dot{\boldsymbol{q}} + \widehat{\mathbf{G}}_{jo} - \widehat{\mathbf{J}}_{c,jo}^{T} \widehat{\boldsymbol{w}}_{c},  \end{array} $$

with *jo* indicating the *n* lower rows of the corresponding matrices and vector, which relate to the actuated joints. The hats on top of the matrices and contact wrench indicates these are now relying on estimated values (dynamic parameters) and the measured state vector. This way of compensating the non-linear dynamics is a form of feedback linearisation [36, 37], also called computed torque-like control [15, 38]. The gain *β*_*in*_ is introduced to reduce the inertial effects in the exoskeleton dynamics compensation control for stability reasons. A complete compensation of the inertial terms of the exoskeleton is not feasible, due to modelling errors and delays. These delays are unavoidable when working with derivatives of measured variables, except when dealing with quasi-cyclic motions [39, 40]. Therefore, the *β*_*in*_ gain is held constant between 0 and 1, close to 1. The gravitational term has no such gain, as it is less prone to modelling errors and does not depend on derivatives of joint angles. When the exoskeleton also has to provide assistance the desired spring torque becomes:

6 $$\begin{array}{*{20}l} \boldsymbol{\tau}_{des,spr} = & \boldsymbol{\tau}_{dyn} + \boldsymbol{\tau}_{assist}.  \end{array} $$

Torque control of SEAs often relies on position (and velocity) controllers at the actuator level [41–43]. Hence, the desired spring torque is converted into a desired spring angle using the torque angle relation:

7 $$\begin{array}{*{20}l} \tau_{spr,i} = f_{i}(\alpha_{i}) \Rightarrow \alpha_{des,i} = f_{i}^{-1} (\tau_{spr,i}),  \end{array} $$

with *f* being the non-linear torque-angle relation. The spring characteristic *f*_*i*_ only depends on joint *i*. These inverse spring calculations are represented by:

8 $$\begin{array}{*{20}l} \boldsymbol{\alpha}_{des} = F_{inv}(\boldsymbol{\tau}_{des,spr}).  \end{array} $$

The spring angle is then enforced by a closed loop position controller. The desired motor angle for each joint is the sum of the spring deflection and the measured joint angle: $\theta _{des,i} = \alpha _{des,i} + \widehat {\phi }_{i}$, or in vector notation:

9 $$\begin{array}{*{20}l} \boldsymbol{\theta}_{des} = \boldsymbol{\alpha}_{des} + \mathbf{S} \widehat{\boldsymbol{q}},  \end{array} $$

with the hat indicating a measured variable and with the selection Matrix **S** only taking the *n* joint angles *ϕ*_*i*_ from the state vector ***q***: [*ϕ*_1_ … *ϕ*_*n*_]^*T*^=**S*q***.

### Environment Contact Modelling

Any contact with the environment imposes constraints on the kinematics in Eqs. (3-4) and results in constraint forces and moments ***w***_*c*_ on the dynamics in Eq. (1). Jacobian **J**_*c*_ represents information about the nature and location of the ground contact. This contact information is derived from sensor data, the exoskeleton kinematics and a model of the environment.

Suitable sensors to detect contact locations are contact switches, pressure sensors, force/torque sensors, or even distance sensors. When none of these sensors are available, at least one absolute orientation measurement from an Inertial Measurement Unit (IMU) is needed to estimate the contact information. Using the exoskeleton kinematics and some mild assumptions about the ground, for example flat surface, the absolute orientation measurement allows to estimate the position and angle of the feet with respect to the ground. This, in turn, allows to estimate the contact information.

The contact wrenches ***w***_*c*_ can be obtained from either measurement or estimation. The first method uses force cells to directly measure the forces and moments. Although these sensors are capable of accurately measuring the wrenches, they have several disadvantages: they need to be placed at all contact locations, they need to be calibrated, and are expensive when high accuracy and repeatability is required. Overloading force cells should also be avoided. Often when a force cell is able to be overloaded more, its resolution is lowered. In the exoskeleton used to validate the developed control methods, no force sensors are available. However, the springs in the SEAs can be seen as simple force cells that allow to measure joint torques. The second method estimates the contact wrenches using the floating base part of the dynamics given by the top three equations of Eq. (1) and the constraint equations (3) and (4). These equations enable us to solve three contact wrench components. Whenever the number of wrench components *k* exceeds three, e.g. when both feet are on the ground, the contact wrenches cannot be solved unambiguously and a contact wrench distribution needs to be found. In humanoid robotics, this is solved by minimizing a weighted combination of contact forces and joint torques [26, 27]. In this paper, we use a similar method [44] in which the weights of the minimization are determined by the stiffness of the exoskeleton parts.

Although contacts are generally modelled with a contact Jacobian and a contact wrench, some contact situations allow alternative modelling approaches. Alternative modelling is required when the contact wrench is hard to obtain or the contact Jacobian cannot be constructed. The contact between an exoskeleton and a chair/stool in a sit-to-stand (STS) motion is a contact that is hard to model and measure. In this case, the stool is supporting the weight of the exoskeleton, reducing the load on the leg actuators. Hence, the stool contact can be modelled by setting the gravity part of the dynamics to zero. When the exoskeleton leaves the stool, the gravity part is again active. This alternative modelling is achieved by adding a changing gravity gain scalar *β*_*g*_ to the exoskeleton dynamics compensation joint torques in Eq. (5):

10 $$\begin{array}{*{20}l} \boldsymbol{\tau}_{dyn} = \beta_{in} \left(\widehat{\mathbf{M}}_{jo} {\ddot{\boldsymbol{q}}} + \widehat{\mathbf{C}}_{jo} {\dot{\boldsymbol{q}}} \right) + \beta_{g} \widehat{\mathbf{G}}_{jo} - \widehat{\mathbf{J}}_{c,jo}^{T} \widehat{\boldsymbol{w}}_{c},  \end{array} $$

where the gain *β*_*g*_ lies between 0 and 1.

### Robot Dynamics in feedforward

This way of compensating the non-linear dynamics is a form of feedback linearisation [36, 37], also called computed torque-like control [15, 38].

Computed torque-like control [38] is a form of feedback linearisation [36, 37]. It relies on a good torque control loop in order to compensate the non-linearities and achieve linear error dynamics. SEAs allow to convert a torque control to a position control problem using Eq. (7). However, feedback position control of the SEAs is insufficient to compensate the exoskeleton dynamics, since the bandwidth of this position controller is always lower than the underlying motor velocity or current control loop. Moreover, the varying inertia (and stiffness) seen by each actuator requires conservative tuning of the feedback gain, which further limits the bandwidth of the position control loop. Therefore, feedforward is essential in SEA control to reduce the torque tracking errors in case of rapidly changing torques.

In order to be as effective as possible, the feedforward signal should enter at the lowest possible level in the actuator controller. This *actuator control level* consists of a current control loop inside the velocity control loop. Current feedforward is directly related to the joint torques, because of the linear relationship between torque and current for electric motors, given by the motor constant *K*_*T*_ (neglecting magnetic saturation). This feedforward also depends on the efficiency of the motor and gear *η*, which is not a constant, and the transmission ratio of the gears, *t*_*r*_. The current feedforward *I*_*ff*_ is based on the work of Paine et al. [45], but extended using Eq. (2) to also take the motor inertia **B**_*ii*_ into account to give for actuator *i*:

11 $$\begin{array}{*{20}l} I_{ff,i} = \frac{\tau_{motor,i}}{(t_{r} \cdot \eta \cdot K_{T})_{i}} = \frac{\tau_{spr,i} + \mathbf{B}_{ii} \ddot{\theta}_{i}}{(t_{r} \cdot \eta \cdot K_{T})_{i}}.  \end{array} $$

If the actuator controller allows no access to the current control loop, the low-level controller needs to provide a feedforward signal on velocity level. Velocity feedforward requires a time derivative of the desired spring deflection angle *α*. By taking the time-derivative of the torque-angle characteristic in Eq. (7):

12 $$\begin{array}{*{20}l} \frac{d \tau_{spr,i}}{dt} = \frac{\partial f_{i}}{\partial \alpha_{i}} \cdot \frac{d \alpha_{i}}{d t},  \end{array} $$

and combining this in a diagonal stiffness matrix **K**(*α*)=*diag*(*∂f*_*i*_/*∂α*_*i*_), the motor velocity feedforward is written as:

13 $$\begin{array}{*{20}l} \dot{\boldsymbol{\theta}}_{ff} = \mathbf{S} \dot{\boldsymbol{q}} + \mathbf{K}^{-1}(\alpha) \dot{\boldsymbol{\tau}}_{des,spr}.  \end{array} $$

The derivative of the spring torque $\dot {\boldsymbol {\tau }}_{des,spr}$ is computed by deriving the desired spring torques in Eq. (6), in which the exoskeleton dynamics compensation torque for STS of Eq. (10) are substituted:

14 $$\begin{array}{*{20}l} \dot{\boldsymbol{\tau}}_{des,spr} =& \beta_{in} ({\widehat{\dot{\mathbf{M}}}}_{jo} \ddot{\boldsymbol{q}} + \widehat{\mathbf{M}}_{jo} \dddot{\boldsymbol{q}} + {\widehat{\dot{\mathbf{C}}}}_{jo} \dot{\boldsymbol{q}} + \widehat{\mathbf{C}}_{jo} \ddot{\boldsymbol{q}}) + \beta_{g} {\widehat{\dot{\mathbf{G}}}}_{jo}  \\ & + \dot{\beta}_{g} \widehat{\mathbf{G}}_{jo} - {\widehat{\dot{\mathbf{J}}}}_{c,jo}^{T} \widehat{\boldsymbol{w}}_{c} - \widehat{\mathbf{J}}_{c,jo}^{T} {\widehat{\dot{\boldsymbol{w}}}}_{c} + \dot{\boldsymbol{\tau}}_{assist}.  \end{array} $$

The rate of change of gain *β*_*g*_ has an important influence on the feedforward signal, as the gravity is multiplied by the time derivative of the gain terms $\dot {\beta }_{g}$, which results in a large contribution to the feedforward signal, $\dot {\beta }_{g} \mathbf {G}_{jo}$. Hence, the influence of changing parameters in the dynamics can be an important contribution to the feedforward command, as shown by the derivative of the spring torque in Eq. (14). The gain for the inertial parameters *β*_*in*_ is a constant and hence no time derivative is needed.

When no desired trajectory is imposed, the exoskeleton needs to follow the human as closely as possible. In this case, the inputs of the velocity feedforward of the low-level controller are based on *measured* state variables and their derivatives: $\widehat {\boldsymbol {q}}, {\widehat {\dot {\boldsymbol {q}}}}$ and ${\widehat {\ddot {\boldsymbol {q}}}}$. To prevent instability due to modelling errors and noise, the feedforward gain needs to be set (slightly) smaller than 100% and the third order derivatives of the measured state vector ${\widehat {\dddot {\boldsymbol {q}}}}$ are omitted, because of their noise sensitivity. Only when working with predefined trajectories or predictions of the desired feedforward, the ***q****...* term is usable when these signals are derivable at least three times. When no desired trajectory is at hand, the exact (full) feedforward cannot be attained in practice.

## Sit-to-stand Exoskeleton

**Fig. 5:**
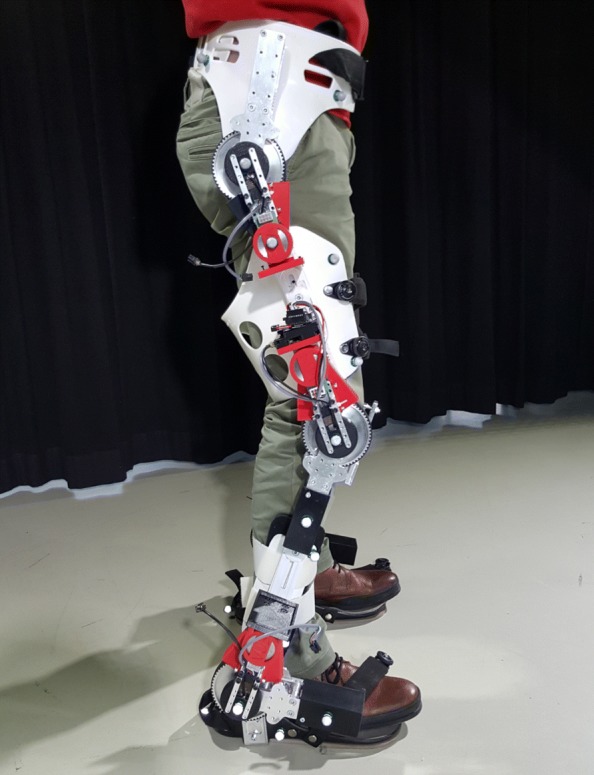
The MIRAD sit-to-stand exoskeleton. A side view of the MIRAD sit-to-stand exoskeleton is shown. The power and communication cables for each joint are not present to avoid overload of the photograph. The MACCEPA actuators are placed at the hip, knee and ankle of a commercial lower limb brace

The contributions of this paper are validated on a bilateral lower-limb exoskeleton for STS, shown in Fig. 5, developed in the MIRAD project. The design of the MIRAD exoskeleton focuses on the hip, knee and ankle joints in the sagittal plane [46]. The range of motion and torques of these joints are largest in the sagittal plane for most activities of daily life. Standing up and sitting down demand high hip extension and knee extension power [47], while the range of joint motion during this activity is large. On the other hand, walking demands high ankle plantarflexion power. Therefore, the exoskeleton only has rotational joints corresponding to the sagittal hip, knee and ankle joints. These joints are equipped with MACCEPA actuators [48], which are SEAs capable of changing their stiffness, i.e. Variable Stiffness Actuators. The MACCEPA actuator uses linear compression springs with a non-linear lever-arm system, but the spring behavior at the joint level is modelled as a non-linear rotary spring according to Eq. (7) and Fig. 3. As the human joints outside the sagittal plane also exhibit small motions, flexibility of the mechanical frame allows small, out-of-plane motions. This makes the exoskeleton significantly more wearable. Additionally, the links are adjustable in length to increase the range of potential users.

**Fig. 6:**
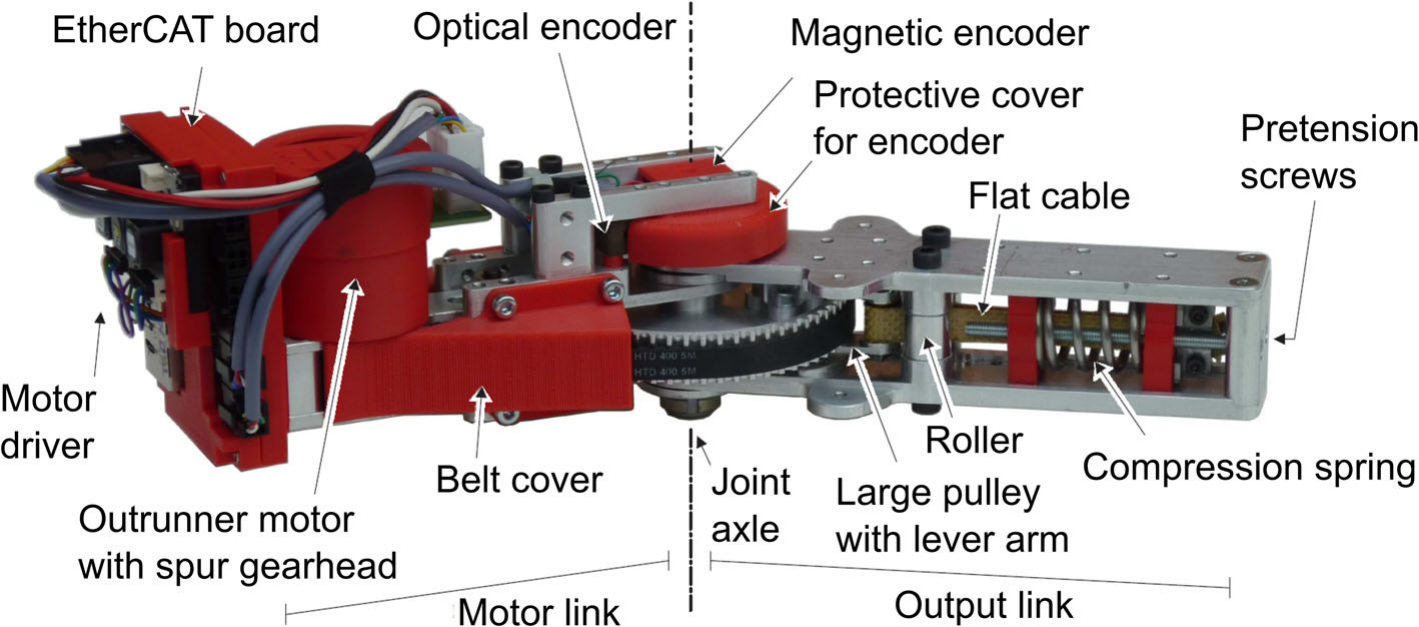
The MACCEPA actuator. The motor is connected to the lever arm with a toothed belt transmission. A flat cable connects the lever arm with the compression spring located in the moving output link. When the lever arm and output link are aligned, the torque generated by the MACCEPA is zero. The pretension screws allow to give the spring a pretension to change the stiffness relationship of the actuator

The MACCEPA actuator used in the exoskeleton is shown in Fig. 6. The lever arm and flat cable transform the linear translation spring into a non-linear stiffening rotational spring behaviour. In addition, the pretension controls the rotational stiffness. More pretension results in higher rotational stiffness. The pretension is kept the same during all motions. The drawback of the lever arm and cable system is the introduction of backlash around the alignment of the lever arm and output link. The motor in this MACCEPA is a Maxon EC 45 flat 50W motor with a Maxon Spur Gearhead GS 45 A and a toothed belt transmission, resulting in a transmission ratio of 188. The nominal torque and nominal velocity at the output of the toothed belt are 11.2Nm and 28rpm (=2.93rad/s) respectively. The peak torque can reach up to 15Nm when overloading the motor.

The actuator is equipped with an absolute magnetic encoder (AMS, AS5048A, SPI, 6 pins, 5V, 14bits) to measure the lever arm angle with respect to the motor link *θ*_*i*_. A relative optical encoder (US Digital HUBDISK-2-2000-625-IE, module: EM1-2-2000-I, DI/O, 5 pins, 5V) measures the angle of the output link with respect to the motor link *ϕ*_*i*_. The motor velocity is estimated from the hall sensors and also motor current is measured. All motor data and sensor data is captured by the communication board on every joint and send to the central processing unit over EtherCAT [49]. In order to have an additional absolute measure of the floating base states (*p*_*x*_,*p*_*y*_ and *p*_*z*_) and their derivatives, an IMU of Xsens [50] provides accelerations, angular rotations and the orientation of the sensor.

The designed actuators including sensors, motor controller and communication boards are mounted on commercial available braces [46]. The mass, center of mass coordinates and the rotational inertia of each part of the exoskeleton are shown in Table 2. These dynamic parameters are identified in previous work [35].

**Table 2:** Dynamic parameters of the STS exoskeleton with all leg lengths set to *L*=0.42*m*

part	mass	center of mass (x,y) w.r.t. joint	rotational inertia
left foot	1.21*kg*	(0.09217,−0.0690)*m*	0.00849*kgm*^2^
left lower leg	1.88*kg*	(0.1883,0.0109)*m*	0.0105*kgm*^2^
left upper leg	2.53*kg*	(0.2268,0.0088)*m*	0.01529*kgm*^2^
right foot	1.19*kg*	(0.09121,−0.0686)*m*	0.00792*kgm*^2^
right lower leg	1.96*kg*	(0.1826,−0.0067)*m*	0.0118*kgm*^2^
right upper leg	2.47*kg*	(0.2301,0.0152)*m*	0.01568*kgm*^2^
pelvis	1.59*kg*	(0.113,−0.072)*m*	0.0072*kgm*^2^

Most exoskeletons that have assisted sit-to-stand motion focus on paraplegic patients [51] [52] or focus on gravity compensation of the person [53]. Nakamura et al. [53] showed a reduction in human EMG when they assisted with a simplified human model to compensate part of the human weight. The MIRAD exoskeleton is intended for people who still have voluntary motion capabilities, and uses the model-based torque control presented in the Methods section. The modelling procedure in Table 1 is illustrated on the STS activity.

### Modelling Step 1: List sensor data available to the exoskeleton controller

The following measured variables are available to the exoskeleton controller:

- relative rotary encoders measure all joint angles *ϕ*_*i*_,
- the joint angular velocity $\dot {\phi }_{i}$ of each joint is obtained by counting the number of high frequency clock pulses between two encoder pulses,
- absolute encoders measure the lever arm angles *θ*_*i*_,
- hall sensors measure the lever arm angular velocities $\dot {\theta }_{i}$,
- an IMU of Xsens placed at the pelvis returns the floating base angle *ψ*, its derivative $\dot {\psi }$ and the accelerations *a*_*x*_ and *a*_*y*_ at the sensor location, which are related to $\ddot {p}_{x}$ and $\ddot {p}_{y}$,
- a second IMU at the sternum monitors the human trunk.

The kinematic constraints in Eq. (3) and (4) enable us to estimate the floating base states and derivatives from the joint angle measurements. However, small deflections out of the 2D plane and noise on higher derivatives of the joint angles, make the floating base derivatives unreliable. Furthermore, the exoskeleton requires at least one absolute measurement to know its orientation in the world. Therefore, an IMU placed at the pelvis provides this additional information. The IMU measurements are fused together with the floating base estimates based on the kinematic constraints. This is done by considering all the IMU measurements and the kinematic constraint estimates as inputs of a Kalman filter with a constant acceleration motion model. The accelerations of the joint angles $\ddot {\phi _{i}}$ and lever arm angles $\ddot {\theta }_{i}$ are obtained also by relying on a Kalman Filter for each joint with a constant acceleration motion model.

### Modelling Step 2: Determine contact situations

**Fig. 7:**
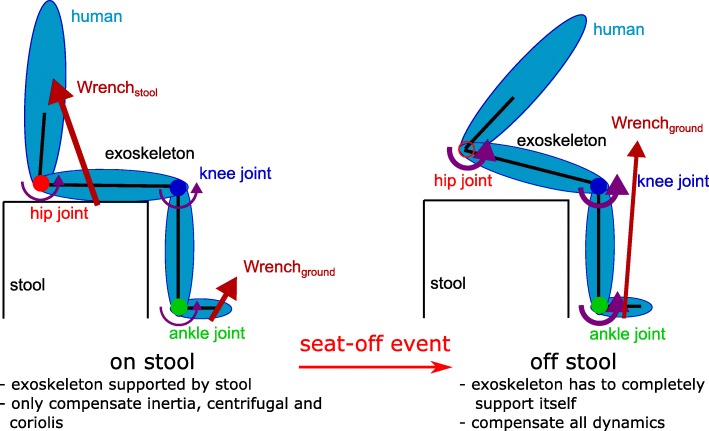
Different contact situation of the sit-to-stand motion. In the sit-to-stand motion, seat-off is the key event for modelling. In the sit state, the exoskeleton is supported by the stool and only the inertial, centrifugal and Coriolis terms are compensated for (only the trunk can move). In the stand state, the exoskeleton is no longer supported by the stool and needs to support its own gravity

The STS activity is characterized by two different contact situations: contact and no contact with the stool, as shown by Fig. 7. In the first case, the exoskeleton has three contacts with the environment: both feet at the ground and the pelvis and upper limbs at the stool. In the second case, only the feet still touch the ground. Seat-off is defined by the instance at which the exoskeleton and person no longer touch the stool and it separates the two contact situations.

**Fig. 8:**
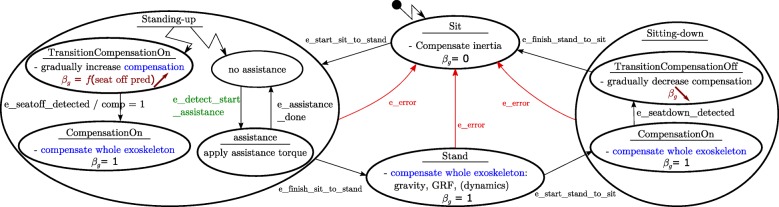
Mid-level controller of the exoskeleton. The Finite State Machine of the sit-to-stand motion has four main states: sit, stand, standing-up and sitting-down. Four events trigger the change from state to state. In the sit state only the inertial and Coriolis compensation of the exoskeleton is active. In the standing-up state, the gravity compensation is gradually turned on before the seat-off. When the compensation gets one or when the seat-off is detected, the gravity compensation is completely on. When the event to start the assistance is detected, the assistance is switched to active. In the stand state the exoskeleton is completely compensated. In the sitting-down state, gravity compensation is gradually reduced to zero until the person sits back on the stool

The two different contact situations are directly related to two discrete states, the sit state and the stand state, in the FSM of the mid-level controller in Fig. 8. The sit state and stand state are accompanied by two additional states for transition between the contact states: a standing-up state and a sitting-down state. The implementation of the action at each state depends on the modelling of the contact situations and follows in step 4. A separate FSM in the standing-up state allows to independently start and stop the assistance. The transition between states is triggered by events, as shown by the arrows in Fig. 8.

### Modelling Step 3: Detect and predict contact transitions

The events that change the state of the FSM are presented in Table 3. All these events are monitored or predicted by the high-level controller. The start of the STS motion is detected by looking at the velocity of the hip angle. When this hip velocity exceeds 14.3 deg/*s* (0.25 *rad*/*s*) the STS motion starts. Other events are explained in Table 3. The seat-off event is predicted using statistical methods and is described in more detail in the work of Tanghe et al. [54]. Briefly explained, a statistical model is constructed relying on measurements of hip, knee and ankle angles during STS motions of non-disable persons without exoskeleton. During these STS movements in the lab, a force plate underneath the stool measured the ground reaction forces. The force plate data was the ground truth for the seat-off event, but this information is not available for the exoskeleton controller. The hip angles were found to hold most information to predict the seat-off event. A method to detect the start of assistance based on Artificial Neural Networks (ANN) is also presented in their work. All predictions and estimations rely on joint measurements, as sensors to detect contact with the stool are not available. Only the joint angles related to stand *q*_*i,stand*_ for determining the end of the STS motion are tuned for each person individually. This is done by capturing these angles when the person is standing upright before the experiments start. All other parameters are generalized and thus used for all participants.

**Table 3:** High-level controller: event detection/prediction

event	detection/prediction
e_start_sit_to_stand	$\dot {q}_{hip} > 14.3 \deg /s$
predict seat-off time *t*_*so*_	*t@* max(*q*_*hip,predicted*_)
e_seatoff_detected	max(*q*_*hip,buffered*_[*t*])== max(*q*_*hip,buffered*_[*t* + 1])
e_detect_start_assistance	*t*_*so*_−*t*_*current*_<0.11*s* or ANN [54]
e_assistance_done	*τ*_*as*_==0
e_finish_sit_to_stand	*q*_*i*_ close to *q*_*i,stand*_
e_start_stand_to_sit	*q*_*knee*_<−28.6 deg and $\dot {q}_{knee} < -17.2 \deg /s$
e_seatdown_detected	*q*_*knee*_<−68.8 deg
e_finish_stand_to_sit	*q*_*knee*_<−77.3 deg and $|\dot {q}_{knee}| < 5.7 \deg /s$
e_error	any encoder, controller or prediction error

### Modelling Step 4: Model the exoskeleton for each situation

The dynamic model for the exoskeleton is a floating base model in both contact situations. In both cases, we assume that both feet stay in contact with the ground, hence no foot is lifted or moved. The contact Jacobian and contact wrench of the feet are constructed with the contact location at the heel. As each foot is also assumed flat on the ground, the contact wrench of each foot consists of two forces and one moment. These contact wrenches cannot be solved with the three floating base equations alone. An optimization based on the stiffness of the exoskeleton [44] is used to solve the six contact wrench components, as described in the Methods section. As the stool contact is both hard to locate and measure, it is modelled by introducing gain parameters in the dynamics, see Eq. (10). When the exoskeleton and human are seated, the stool supports almost all the weight. Hence, the exoskeleton model is a floating base model without gravity by setting *β*_*g*_ to 0. When the stool contact is broken, the exoskeleton is required to compensate its own weight and the gain *β*_*g*_ is set to 1.

The exoskeleton gravity compensation gain *β*_*g*_ should not be turned on and off instantaneously. From measurements of the human-stool interaction forces we know that the reaction force on the stool decreases almost linearly, starting approximately 0.3s before seat-off until seat-off [54]. Hence, the model of the exoskeleton has to change linearly from a model without gravity at 0.3s before seat-off to a model with full gravity at seat-off, i.e. the exoskeleton gravity compensation gain *β*_*g*_ needs to increase linearly from zero to one in approximately 0.3s. This linear increase of *β*_*g*_ is implemented in the standing-up state in Fig. 8. The increase in *β*_*g*_ is made dependent of the time until the current predicted seat-off: *β*_*g*_=1−*t*_*untillseat*−*off,predicted*_/0.3*s* when *t*_*untillseat*−*off,predicted*_>0.3*s*, with *t*_*untillseat*−*off,predicted*_ is the predicted time until seat-off. This ensures that the exoskeleton gravity compensation is built up faster when the seat-off is reached faster. This shows the importance of the two additional states standing-up and sitting-down in the FSM. The gain *β*_*g*_ used to switch the contact model is shown in the FSM.

### Overview of the low-level controller

**Fig. 9:**
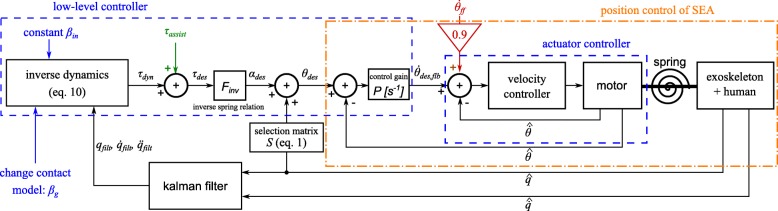
Overview of the exoskeleton controllers. The control loops of the exoskeleton are given. The actuator controller is a velocity controller of the motor angle ***θ***. The input of this actuator controller comes from the low-level controller: the desired velocity and the velocity feedforward. Both signals use the dynamic model and the model of the spring. The feedforward $\dot {\boldsymbol {\theta }}_{ff}$ is obtained from Eq. (13-14)

Figure 9 gives an overview of the continuous control loops for the STS exoskeleton. The focus lies on the actuator and low-level controllers, as the mid-level and high-level controllers are already shown in Fig. 8 and Table 3 respectively. The control loops are shown for an exoskeleton with velocity controlled SEAs. In the low-level controller the dynamic model generates the spring torque ***τ***_*dyn*_ required to compensate the exoskeleton dynamics. The inertial and Coriolis and centrifugal parts of the dynamics are only partially compensated by setting *β*_*in*_ to a constant gain of 0.75. A full dynamic compensation of the exoskeleton is not feasible due to modelling errors, filtering delays, closure of the loop with measured variables and the play in the Maccepa actuators around zero torque. The joints are filtered with independent constant acceleration Kalman filters.

The spring torques of the exoskeleton dynamics compensation ***τ***_*dyn*_ are combined with the assistance torques ***τ***_*assist*_ to give the desired joint torques ***τ***_*des*_. These torques ***τ***_*des*_ are transformed to desired motor angle ***θ***_*des*_ inputs for each actuator controller, using Eqs. (7-9) in the low-level controller. This is followed by a closed-loop proportional position control (orange) with a gain *P* of 10*s*^−1^, which results in a position bandwidth around 1.6Hz. This low gain keeps the system conservatively stable under the changing inertia of the non-linear exoskeleton dynamics and under the non-linear spring. To make up for the low position bandwidth, velocity feedforward $\dot {\boldsymbol {\theta }}_{ff}$ is introduced using Eqs. (13-14). The signal enters the velocity control loop as the motor current control is not accessible. The feedforward, which relies on measured variables in this STS motion, is set to 90%, to prevent instability. Also, the third order derivative of position ***q****...* of Eq. (14) is not considered due to the high influence of noise on higher derivatives.

## Experiments and Results

We validated the exoskeleton dynamics compensation controller on non-disabled individuals. If the model-based compensation of the exoskeleton dynamics is successful, there should be no difference between muscle effort during STS when no exoskeleton is worn and when the exoskeleton, controlled for near-full (*β*_*in*_= 0.75) exoskeleton dynamics compensation, is worn. Ideally, this compensation of the exoskeleton dynamics should be 1 (100%), but could not be attained in practise. Also the human kinematics and dynamics should be similar in both cases. In addition, we also verified how well the torque controller was able to deliver the demanded spring torque. The stand-to-sit motion was not analysed in depth, because we focused on standing up. No walker or any other support was used during the STS experiments.

### Participants

The exoskeleton was tested on 15 young adults (10 males, 5 females, 26.4±3.3 years, 71.3±9.2*kg*). The participants had no prior experience with exoskeletons. The exoskeleton link lengths were adjusted to each participant and the exoskeleton joints were aligned with the participant’s joints. All subjects gave informed consent in accordance to the ethical committee of UZ Leuven before participating in the study.

### Assistance

The provided assistance torques are additional torques in addition to the exoskeleton dynamics compensation torques. As the non-disabled participants do not need this assistance, the goal of the assistance is to reduce the contribution of the participants compared to the case when no assistance is given. The assistance is an off-line computed assistance-as-needed (AAN) torque. Hence, this was a precomputed torque profile that was given as assistance in the assistance-as-needed condition. The start of the off-line generated assistance was learnt using artificial neural networks [54]. These neural networks learned to map the start of assistance on the human torques needed during standing up. The network was trained on data from the initial tests on several volunteers to only predict when the off-line generated assistance needed to start. Training the network on several persons allowed to use the network on multiple participants without having to calibrate for every individual. However, when the motion deviates a lot from the demonstrated motions (elderly or disabled), the neural network needs retraining.

To compute this assistance, data from an earlier study [30] was used. Marker data of several participants of this study was used to scale musculoskeletal models in OpenSim 3.2 [55]. To simulate muscle weakness, the muscles were artificially weakened in the musculoskeletal models. The same kinematics and force plate data was applied to the models. Because of the muscle weakening, the models were no longer able to generate the required torques to perform the recorded motion in simulation. Subtracting the simulated muscle-generated torques from the required torques, prescribed by the human dynamics, leads to the assistance-as-needed torques. Eventually, this should be calculated on-line while moving with the exoskeleton. However, in these first experiments, these calculation were simulated off-line and averaged over the seven non-disabled participants of the earlier study.

### Protocol

Each participant came once to the measurement lab for the full experimental protocol. The measurement involved standing up from and sitting down on a stool, set to 100% knee height. Each STS movement (with or without exoskeleton) was characterized as follows. The participant was instructed to wait for a verbal signal before standing up. After the verbal signal the participant could choose freely when to stand up at a self selected speed, but was requested to do so fluently. After the participant was standing, he was instructed to sit down after approximately three seconds.

Four different conditions were analysed: condition **without exoskeleton N**, **passive P** condition (exoskeleton is unpowered), **transparent T** condition (exoskeleton is compensated, no assistance) and off-line **assistance-as-needed A** condition, always performed in that order. The passive condition was evaluated to Because adjusting the exoskeleton to the participant and putting on the exoskeleton takes almost half an hour, the motions without exoskeleton were always performed first. The participant had to stand up and sit down five times in condition **N**. Next, the exoskeleton was put on and the exoskeleton IMU sensors were calibrated. For each exoskeleton condition, 21 motions were performed, of which the first, middle and last three were recorded. This way, we could analyse if the user would require some time to adapt to the control method in each condition. However, a required adaptation time was not perceived. In this paper, only the last three motions of each condition were used. Between each exoskeleton condition, a short break of 5min. was introduced to prevent fatigue and to let the participant fill out a questionnaire.

### Kinematics and kinetics

To validate the exoskeleton controller, the motions of the participant and exoskeleton were recorded with a reflective marker motion capture system consisting of ten cameras (Vicon Motion Systems, Oxford Metrics, UK) sampled at 100Hz. A total of 34 markers were placed on bony landmarks of the participant. In addition, clusters with three markers were placed on lower and upper arms and upper legs, and a head band with four markers was worn by the participant. In the exoskeleton condition, the markers on the participant’s pelvis were removed due to incompatibility with the exoskeleton pelvis module. Four markers placed on the exoskeleton pelvis module were used to locate the participant’s pelvis.

Three force plates (AMTI, Waterworn, USA) placed under both feet and a stool respectively captured the ground reaction forces at a sample frequency of 1000Hz. The way the force plates were set up in the lab prevented the participants from placing their feet in a self-selected position. As a result, the right leg was placed slightly more outward compared to a self-selecting *normal* sitting position. Attention was paid to make sure the feet were placed in the same positions in every condition. Placing the feet in a non self-selected position might have an impact on the kinematics, torques and muscle usage. Because the foot placement was kept the same for every condition, any deviation due to a non self-selected foot placement is present in every measured condition. In addition, the data used for the off-line assistance torques [30] was also recorded with the same foot placement.

All marker data and force plate data were low-pass second order Butterworth filtered with zero-phase lag at 6Hz. Inverse kinematics of the marker data resulted in the joint kinematics using OpenSim 3.2. A participant model was created by scaling the standard OpenSim generic_92_23 model to the weight and size of the participant. The exoskeleton dynamic model was added to this scaled participant model. The inverse dynamics were calculated in OpenSim 3.2 using this total model, the calculated joint kinematics and force plate data. This resulted in the total joint torques of both participant and exoskeleton. The marker and force plate data were synchronized with the exoskeleton data, which was recorded at 500Hz. This allowed to subtract the exoskeleton torque contribution (Eq. (7)) from the total joint torques to give the participant’s joint torques.

### Electromyography

The different control conditions were also compared by looking at muscle usage. We measured surface electromyography (EMG) signals of the rectus femoris (RF), vastus lateralis (VL), biceps femoris (BF), medial hamstrings (MH), tibialis anterior (TA), gastrocnemius lateralis (GL), soleus (So) and vastus medialis (VM) bilaterally. The self-adhesive electrodes were placed two centimetres apart and in the fibre direction on the belly of each muscle in accordance with the Seniam guidelines [56]. EMG signals of the 14 muscles were sampled at 1000 Hz using a wireless EMG system (ZeroWire, Aurion). The raw EMG signals were band pass filtered between 20 and 400Hz with a second order Butterworth filter. The signal was then rectified and low pass filtered at 10Hz with a second order Butterworth filter. The EMG processing is done according to the Seniam guidelines [56] The muscle effort was approximated by the time integral of the rectified and filtered EMG values during the STS motion. Since there was a resting period between the measured conditions, the EMG was not corrected for fatigue between conditions.

### Statistics

The relative change in muscle effort in the passive condition compared to the transparent condition and in the condition without exoskeleton compared to the transparent condition was evaluated with a Wilcoxon Signed Rank Test [57]. The statistical tests of the 14 muscles are one family of tests. Hence, p-values of the muscles tests were corrected for multiple comparisons with Bonferroni-Holm [58] correction. The 14 p-values from comparing two conditions are ordered from low to high. The p-value on rank *i* is Bonferroni corrected for 14−*i*+1 tests (*p*_*Bonferroni,i*_=(14−*i*+1)×*p*) and compared to the significance level (here 0.05). When the Bonferroni corrected p-value of rank *i* is not significant, the test stops and all following p-values (*i*+1 to 14) are also not significant. There are three families of muscle tests: passive, transparent and without exoskeleton. The multiple testing of the three conditions is corrected by a simple Bonferroni correction by multiplying all p-values by 3.

To compare the human joint angles and torques between the different conditions, a one dimensional Statistical Parametric Mapping (SPM) Friedman’s ANOVA was used [59]. This method also corrects for multiple tests. When significance was reached, a post-hoc test was needed to find between which groups the differences were significant. This is done with a two-sample t-test between the combination of conditions N vs T and P vs T, again correcting for multiple testing.

### Results

**Fig. 10:**
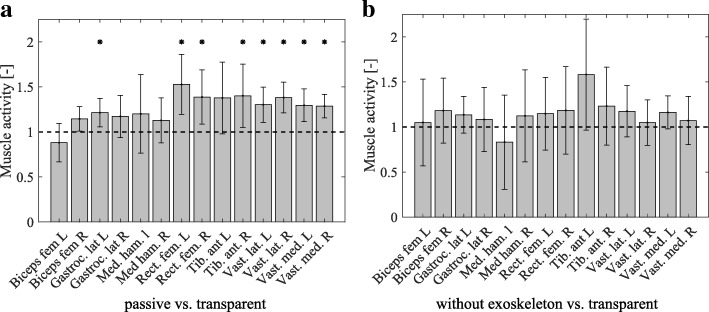
Comparison of muscle effort in three conditions: passive, transparent and without exoskeleton. The average muscle effort (time integral of the muscle activity) is shown relative to the transparent condition by the gray bars and the corresponding standard deviation across all fifteen participants is shown by the error bars. Each muscle is scaled separately relative to the corresponding average muscle effort in the transparent condition. The black dotted line represents this relative muscle activity in the transparent condition, scaled to one. In the left subfigure (a) the muscle effort of the passive condition is compared to the transparent condition. In the right subfigure (b) the effort of the condition without exoskeleton is compared to the transparent condition. When the plotted bars and error bars lie above one, the muscle effort is larger than in the transparent condition. Significant differences between the without exoskeleton and transparent condition are marked with a black star (*p*_*Bonferroni*−*Holm*_<0.05)

The main objective of the experiments was to show the effectiveness of the model-based compensation of the exoskeleton dynamics. Therefore, the muscle efforts of fourteen muscles were compared in Fig. 10. Especially the quadriceps muscles, of which the vastus medialis, vastus lateralis and the rectus femoris were measured, contribute to the STS motion. Eight out of the fourteen muscles required significantly (Bonferroni-Holm corrected p <0.05) more effort in the passive condition P relative to the transparent condition T, as Fig. 10a and Table 4 show. All of the recorded quadriceps muscles (RF, VL and VM) showed significant difference. In contrast, in the muscle effort of the condition without exoskeleton N compared to condition T, no significant differences were detected in any muscle, as Table 5 and Fig. 10b show.

**Table 4:** Bonferroni-Holm corrected p-values P vs T

left muscle	corrected p-value	uncorrected p-value	right muscle	corrected p-value	uncorrected p-value
BF L	0.7632	0.1272	BF R	0.0945	0.0052
GL L	0.0293	0.0012	GL R	0.3926	0.0327
MH L	0.7632	0.1272	MH R	0.4449	0.0494
RF L	0.0051	0.0001	RF R	0.0293	0.0012
TA L	0.1465	0.0098	TA R	0.0081	0.0002
VL L	0.0081	0.0002	VL R	0.0051	0.0001
VM L	0.0073	0.0002	VM R	0.0048	0.0001

**Table 5:** Bonferroni-Holm corrected p-values N vs T

left muscle	corrected p-value	uncorrected p-value	right muscle	corrected p-value	uncorrected p-value
BF L	1	1	BF R	1	0.1040
GL L	1	0.0494	GL R	1	0.3757
MH L	1	0.0923	MH R	1	0.6355
RF L	1	0.2412	RF R	1	0.1909
TA L	0.5333	0.0137	TA R	1	0.1272
VL L	1	0.0419	VL R	1	0.1531
VM L	0.3384	0.0081	VM R	1	0.0676

**Fig. 11:**
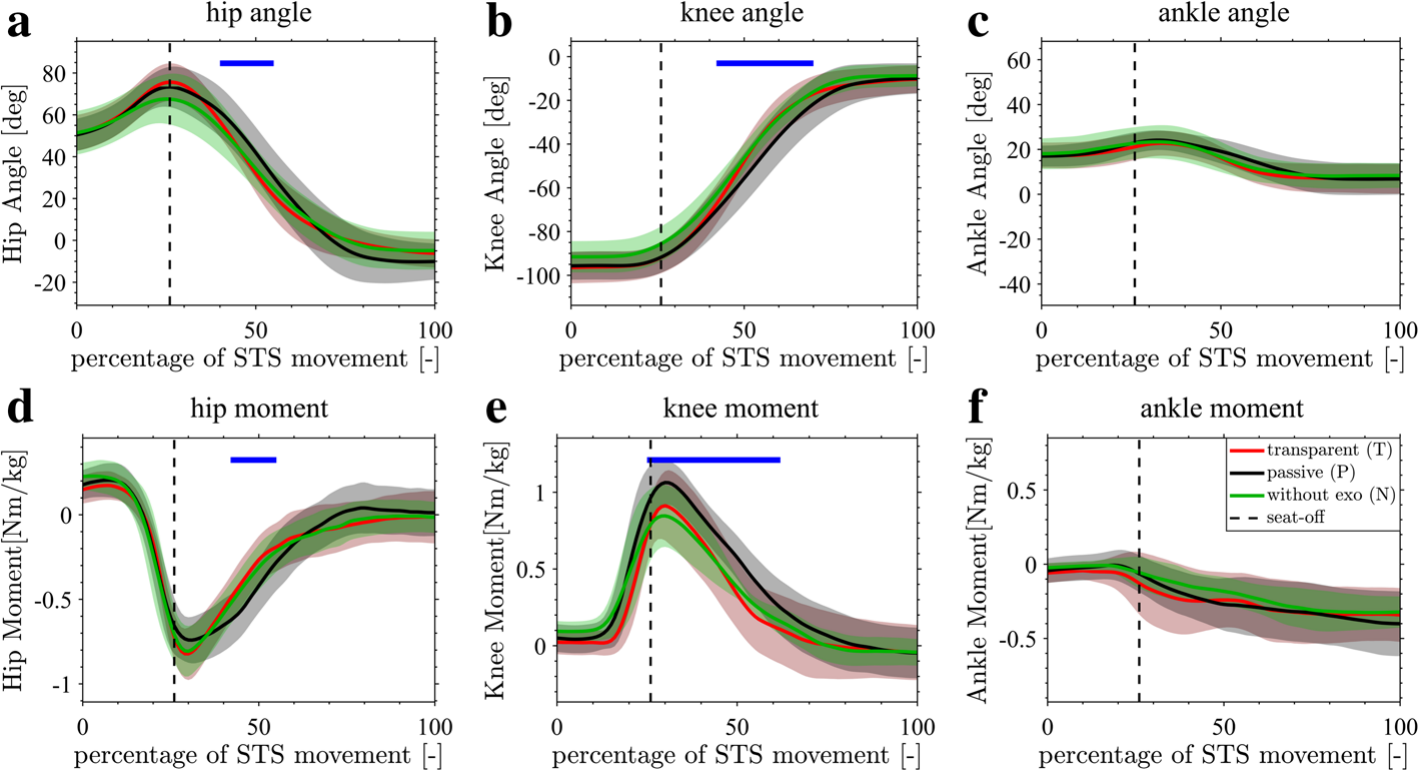
Comparison of human joint kinematics and dynamics in three conditions: passive, transparent and without exoskeleton. The human kinematics and joint torques of the hip, knee and ankle are shown in three conditions: without exoskeleton (N) in green, the transparent exoskeleton condition (T) in red and the passive exoskeleton condition (P) in black. The top three subfigures (a to c) represent the joint angles, while the lower three subfigures (d to f) represent the joint torques, normalized with respect to the mass of each participant. Both the average and standard deviation (band of ±*σ* are shown in each graph. The blue bars indicate time periods with a significant difference between the passive and transparent condition of the post-hoc two-sample t-test. The corrected p-values are 0.008, <0.001, 0.007 and <0.001 for the hip angle, knee angle, hip torque and knee torque respectively

To further examine the effectiveness of the transparent control, we compared human kinematics and joint torques of the hip, knee and ankle during the STS motions computed by SPM in Fig. 11. The reference posture for the human joint kinematics (Fig. 11a to 11c) is standing upright (zero angles). The comparison of kinematics between the transparent condition T in red and the passive condition P in black showed significant differences (*p*_*corrected*_<0.05) in the hip (*p*_*corrected*_=0.008) and knee (*p*_*corrected*_<0.001) angle around 50% of the STS motion, indicated by a blue bar. This could be expected as in condition P, the hip (more flexion) and knee angle (more flexion) evolved slower to the angles of standing upright. No significant differences (*p*_*corrected*_>0.05) were found in the kinematics between the condition T in red and condition without exoskeleton N in blue during STS. The human joint torques (Fig. 11d to 11f) showed significant differences (*p*_*corrected*_<0.05) between condition P and T in the hip (*p*_*corrected*_=0.007) and knee torque (*p*_*corrected*_<0.001), indicated by a green bar. In condition P, the external hip torque was more negative (more flexion torque) from 40 to 50% of the motion, while the external knee torque was significantly larger (more flexion torque) from 30 to 65% of the STS motion. No significant differences (*p*_*corrected*_>0.05) were found in the hip, knee and ankle for condition T and condition N, during the STS motions.

**Fig. 12:**
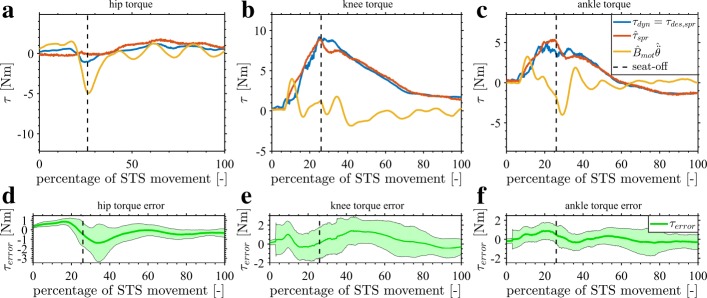
Exoskeleton transparent control evaluation. The joint torque analysis for the transparent condition is shown (condition T). The top three subfigures (a to c) show the joint torque for the hip, knee and ankle for one STS motion. The desired joint torque ***τ***_*des,spr*_ is shown in blue. This desired spring torque is torque needed to compensate the exoskeleton. The measured spring torque $\widehat {\boldsymbol {\tau }}_{spr}$ is represented in red. The torque to accelerate the motor inertia is given in yellow. Seat-off occurs at the black dashed line. The lower three subfigures (d to f) show the average error (difference) and the band of standard deviation (±*σ*) between the desired ***τ***_*des,spr*_ and the delivered $\widehat {\boldsymbol {\tau }}_{spr}$ spring torques for all the STS motions of condition T

As the focus is on the compensation of the exoskeleton, Fig. 12 shows the effectiveness of the torque control loop. The desired ***τ***_*des,spr*_ and measured $\widehat {\boldsymbol {\tau }}_{spr}$ spring torques of the hip, knee and ankle joints are compared in the transparent control condition T. The controller is able to track the desired ***τ***_*des,spr*_ spring torques. This is also confirmed by the low torque errors $\boldsymbol {\tau }_{error} =(\boldsymbol {\tau }_{des,spr} - \widehat {\boldsymbol {\tau }}_{spr})$ in Fig. 12d to 12f.

**Fig. 13:**
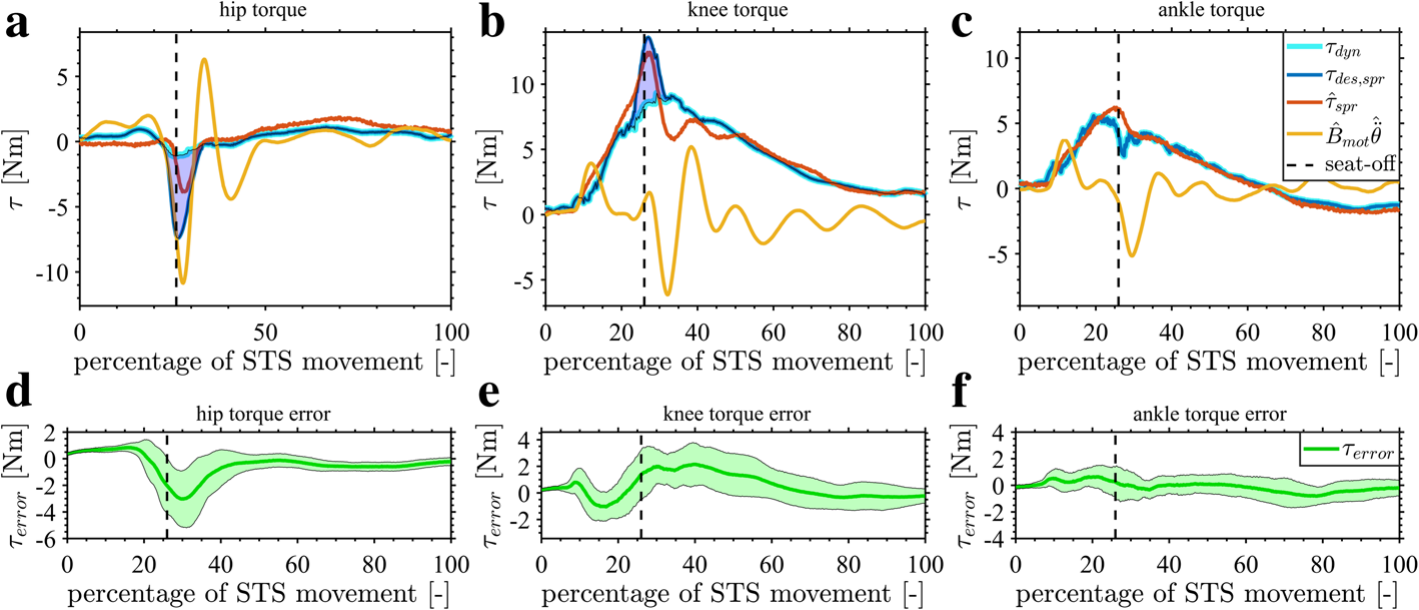
Exoskeleton joint torque control evaluation. The joint torque analysis for the offline assistance-as-needed is shown (condition A). The top three subfigures (a to c) show the joint torque for the hip, knee and ankle for one STS motion. The desired joint torque ***τ***_*des,spr*_ is shown in blue. This desired spring torque is the sum of the exoskeleton dynamics compensation torque ***τ***_*dyn*_, shown by the light blue curve, and the desired assistance indicated by the blue area between ***τ***_*dyn*_ and ***τ***_*des,spr*_. The realised spring torque $\widehat {\boldsymbol {\tau }}_{spr}$ is represented in red. The torque to accelerate the motor inertia is given in yellow. Seat-off occurs at the black dashed line. The lower three subfigures (d to f) show the average error (difference) and the band of standard deviation (±*σ*) between the desired and the delivered spring torques for all the STS motions of condition A

The results of Fig. 13 show the desired and delivered torques at the hip, knee and ankle joint in the assistance-as-needed condition (A). During the largest part of the STS motion, the desired ***τ***_*des,spr*_ in blue and measured (=delivered) $\widehat {\boldsymbol {\tau }}_{spr}$ in red spring torque overlap in Fig. 13a to 13c, and the average errors $\boldsymbol {\tau }_{error} =(\boldsymbol {\tau }_{des,spr} - \widehat {\boldsymbol {\tau }}_{spr})$ remain small in Fig. 13d to 13f. However, the measured hip torque $\widehat {\boldsymbol {\tau }}_{spr}$ is lower than the desired one ***τ***_*des,spr*_ around seat-off in Fig. 13a, which means the assistance is not given completely. This is also indicated by the large average error ***τ***_*error*_ in Fig. 13d. At this location, the motor measured acceleration torque $\widehat {\mathbf {B}}_{mot} {\widehat {\ddot {\boldsymbol {\theta }}}}$ (in orange) of the hip is very high leading to saturation of the hip motor. In contrast, the knee torque is able to follow the desired torque around seat-off, which means the assistance is given completely. Important to note is that the required steep build-up of torque at the knee before seat-off is achieved by the controller.

## Discussion

In order to compensate the exoskeleton dynamics, modelling the contacts of the exoskeleton with the environment are crucial. These contacts play a major role in the controller, as they largely affect the exoskeleton dynamics. Modelling these contacts is often not tackled but evaded by utilizing different dynamic models for every contact situation [14] or changing the control strategy [1, 7]. In this work, effort is put in modelling the contacts and using the same dynamic model (floating base) in the different contact situations.

The STS motion demonstrated the importance of good contact modelling: the stool contact changes, which greatly influenced the exoskeleton dynamics. While on the stool, the exoskeleton is supported and the actuators do not need to compensate the weight of the exoskeleton. The stool contact is modelled by setting the gravity of the exoskeleton to zero with gain *β*_*g*_=0. When the exoskeleton leaves the stool, it needs to compensate its whole dynamics. All the weight of the exoskeleton is transferred to the exoskeleton feet and *β*_*g*_ is set to 1. The inertial term *β*_*in*_ was active in both contact situations. This allowed to also compensate the exoskeleton when the person moves his trunk forward while sitting on the stool. The distribution of the forces over both feet while not on the stool is performed with a model-based method [44] that account for the stiffness of the exoskeleton.

Modelling all contact situations and relying on a floating base model makes the model-based exoskeleton compensation easier generalizable to other contact situations. Modelling the contact results in a limited number of gain factors to capture the change of the dynamics. Only one gain factor *β*_*g*_ models the stool contact in the two situations. The prescribed variation of *β*_*g*_ is directly linked to the change of the vertical ground reaction force when the stool contact changes [54]. The difference with prescribing joint trajectories in impedance controllers [3, 4] is that *β*_*g*_ is not a motion trajectory, but it is one parameter modelling the time variation of the gravity term of the exoskeleton. Furthermore, prescribing *β*_*g*_ is no longer needed when a low-cost pressure sensor would track the change of contact pressure at the stool. The change in pressure could be directly mapped on *β*_*g*_.

In the passive condition (P), the human needed to move the exoskeleton as the controller was not actuating the exoskeleton motors. This is validated by a significant increase in hip and knee flexion torque to stand up in condition P, compared to transparent condition T (see Fig. 11). Especially the human knee torque was larger in condition P, because the exoskeleton needed larger knee torques (see purple dashed line in Fig. 13b). The analysis of muscle effort backs up this increase in joint torque in condition P. The quadriceps muscles, of which VM, VL and RF were measured, were significantly (Bonferroni-Holm corrected p <0.05) more recruited in condition P compared to condition T, as seen in Fig. 10a and Table 4. The significant difference in joint kinematics between condition P and T around 50% of the STS motion can be explained by a slower first half of the STS motion in condition P.

In the transparent condition (T), the exoskeleton had to compensate its own dynamics, taking into account the changing stool contact. If the contact was modelled in the wrong way, such that the controller would compensate the exoskeleton too early or too late, significant differences in human joint torques and muscle effort was expected. However, no significant differences (*p*_*corrected*_>0.05) were found for the joint torques or the muscle efforts between condition T and the condition without exoskeleton N. The uncorrected p-values of the muscle effort between conditions T and N are found in Table 5. These values illustrate the non-significance of the differences between N and T. This indicates the contact modelling of the stool using *β*_*g*_ is on time. Although the absence of significant differences does not prove statistical similarity between condition T and N, the human joint torques show great similarity in Fig. 11. Evaluation of the control errors ***τ***_*error*_ in Fig. 12 d to Fig. 12f indicates that the controller is able to track the desired spring torque to compensate the exoskeleton and provide assistance. Hence, the model-based controller seems to be able to compensate the exoskeleton dynamics.

Prior to the seat-off, a steep build-up of knee torque for the exoskeleton dynamics compensation and assistance is demanded in Fig. 13b. This steep build-up of spring torque at the knee would be impossible by the SEA position control loop alone because it has a low bandwidth (see Subsection Overview of the low-level controller). The torque needs to be build up fast as in less than 0.3s after the seat-off prediction the peak torques at the knee have to be reached. The feedforward control command from Eqs. (13-14) is crucial to prevent tracking errors in the spring compression, which would result in less torque generated.

The error between the desired and measured knee torque right after the assistance (Fig. 13b) and the large error at the hip torque around seat-off (Fig. 13a) can be explained by motor saturation. The motor inertial torque $\widehat {\mathbf {B}} {\widehat {\ddot {\boldsymbol {\theta }}}}$ and the measured spring torque $\widehat {\boldsymbol {\tau }}_{spr}$ add up to about 15*Nm* which is the motor torque limit when overloading the motor. The high hip motor acceleration and high motor inertia, due to a high transmission ratio, led to a high motor inertial torque $\widehat {\mathbf {B}} {\widehat {\ddot {\boldsymbol {\theta }}}}$ as shown in Fig. 13a. This shows the importance of the transmission ratio in the design of the exoskeleton. The high motor inertial torque $\widehat {\mathbf {B}} {\widehat {\ddot {\boldsymbol {\theta }}}}$ drained most of the available motor torque, preventing the spring torque $\widehat {\boldsymbol {\tau }}_{spr}$ from reaching its desired value ***τ***_*des,spr*_. The motor saturation did not influence stability. The saturation only prevented to provide proper hip assistance even though the assistance was low (6Nm). When no assistance was needed (in condition T), no motor saturation occurred, as Fig. 12 shows. In order to overcome this saturation limit, the motor and transmission ratio should be redesigned to reduce the reflected motor inertia after transmission and/or to deliver higher torques.

The motor saturation indicates the importance of a proper motor and transmission design in the early design stages. To this end, not only the desired assistance but also the dynamics of the exoskeleton itself needs to be accounted for. Although the exact mechanical design of the exoskeleton might still be unknown in the early design stages, a rough estimate is needed to simulate the motor torques needed to move the exoskeleton based on human kinematics. In addition, the selection of motor and transmission have to be solved concurrently, as the transmission affects both the torque and the motor inertia presented at the joint level. The selected motor and transmission will affect the exoskeleton inertia and thus alter the torques needed to move the exoskeleton. Hence, an iterative design procedure will be needed where motor and transmission selection and exoskeleton dynamics simulation will have to be repeated until convergence to a valid solution.

The high standard deviation on the spring torque error ***τ***_*error*_ in Fig. 13d to 13f is partially caused by the difficulty in defining the end of an STS motion. The STS motion ends when the person is standing upright. The joint angles need to be close to a defined set of angles related to standing and the joint velocities should be low. Because there is a difference in how fast the person executes the last part of the STS motion, the seat-off instance (where for example the hip torque error is largest) is occurring at slightly different normalized times for every participant. Therefore, the time normalized torque error curves show larger variance between subjects.

The study has three main limitations. First, the exoskeleton actuators (motor and transmission) were not able to assist the hip at the required dynamic profile, leading to motor saturation. Although compensation of the exoskeleton dynamics was possible, the motors were close to or at saturation and unable to deliver any higher assistance torques. Because the assistance was small, the assistance condition did not differ greatly from the transparent control control condition. Consequently, comparisons of muscle effort did not result in significant differences between the transparent and assisted conditions. These analysis are left out of the paper. Second, the control method was not directly compared to the impedance controller, which is a commonly used method in the literature. Such an impedance controller imposes a desired trajectory, which is difficult to provide in non-cyclic motions and while keeping the user in charge. Also, the detailed implementation of the impedance controllers is not documented in depth and these controllers have more gains that require tuning, which prevents an proper comparison. The third limitation is that only sit-to-stand motions were examined. In order to further validate the controller methodology to compensate the exoskeleton and model contacts with the ground, other tasks, such as walking, should be examined in the future.

## Conclusion

The present study proposed a model-based control to compensate the dynamics of a lower-limb exoskeleton. This control approach was tested and evaluated on an exoskeleton for sit-to-stand motions.

Compensating the dynamics of exoskeletons becomes important when they enter daily life where the user wants to be in charge. Controllers capable of compensating the exoskeleton rely on extensive modelling the exoskeleton and the contacts with the environment. These contacts play a major role in this controller, as they largely affect the exoskeleton dynamics. The sit-to-stand motion is a good example to demonstrate the importance of good contact modelling: the stool contact changes, which greatly influenced the exoskeleton dynamics. While on the stool, the exoskeleton is supported and the actuators do not need to compensate the weight of the exoskeleton. When the exoskeleton leaves the stool, it needs to compensate its whole dynamics.

To aid in developing such exoskeleton dynamics compensation controllers, a modelling procedure and control scheme are presented in this work. The control scheme breaks down the controller in four different control levels in support of the modelling steps. Starting from the dynamic model of the exoskeleton, the exoskeleton compensation controller is designed. The outputs of the exoskeleton compensation controller are the exoskeleton compensation torques, which are enforced by a position controlled Series Elastic Actuator. The proposed model-based feedforward signal improves the tracking of the position control loop. A change of the contact model also contributes to the feedforward signal.

The effectiveness of the controller was demonstrated by validating it on non-disabled participants. The controller was able to follow the fast changing torque demands, except when motor saturation occurred (at the hips). Comparing the results between the passive exoskeleton and the transparent controller showed significantly larger human joint torques and more muscle effort for the passive exoskeleton. On the other hand, comparing the transparent controller and the condition without exoskeleton showed no significant difference in results for the human torque and muscle effort.

The saturation of the actuators during the assisted condition, prevented a proper evaluation of the control methods while giving assistance. Future work will include validating the controller on an exoskeleton with more powerful actuators and testing and validating the control methods on walking exoskeletons.

## Abbreviations

A: Assistance-as-needed condition
AAN: Assistance-as-needed
ADL: Activities of daily living
ANN: Artificial neural networks
CAD: Computer aided design
dof: Degree of freedom
EMG: Electromyography
FSM: Finite state machine
IMU: Inertial measurement unit
MACCEPA: Mechanically adjustable compliance and controllable equilibrium position actuator
N: Condition without exoskeleton
P: Passive condition
SEA: Series elastic actuator
STS: Sit-to-stand
T: Transparent condition

## References

[1] Young AJ, Ferris DP (2017). State of the art and future directions for lower limb robotic exoskeletons. IEEE Trans Neural Syst Rehabil Engineer.

[2] Yan T, Cempini M, Oddo CM, Vitiello N (2015). Review of assistive strategies in powered lower-limb orthoses and exoskeletons. Robot Auton Syst.

[3] Kolakowsky-Hayner S, Crew J, Moran S, Shah A. Safety and feasibility of using the ekso bionic exoskeleton to aid ambulation after spinal cord injury. J Spine. 2013;4(3).

[4] Wang S (2015). Design and control of the mindwalker exoskeleton. IEEE Trans Neural Syst Rehabil Engineer.

[5] Jezernik S, Colombo G, Morari M (2004). Automatic gait-pattern adaptation algorithms for rehabilitation with a 4-dof robotic orthosis. IEEE Trans Robot Autom.

[6] Blaya J, Herr H (2004). Adaptive Control of a Variable-Impedance Ankle-Foot Orthosis to Assist Drop-Foot Gait. IEEE Trans Neural Syst Rehabil Engineer.

[7] Jiménez-Fabián R, Verlinden O (2012). Review of control algorithms for robotic ankle systems in lower-limb orthoses, prostheses, and exoskeletons. Med Engineer & Phys.

[8] Aguirre-Ollinger G, Colgate JE, Peshkin MA, Goswami A (2007). Active-impedance control of a lower-limb assistive exoskeleton. Proceedings of the 2007 IEEE 10th International Conference on Rehabilitation Robotics.

[9] Bernhardt M, Frey M, Colombo G, Riener R (2005). Hybrid force-position control yields cooperative behaviour of the rehabilitation robot lokomat. 9th International Conference on Rehabilitation Robotics, 2005.

[10] Duschau-Wicke A, Zitzewitz Jv, Caprez A, Lünenburger L, Riener R (2010). Path control: A method for patient-cooperative robot-aided gait rehabilitation. IEEE Trans Neural Syst Rehabil Engineer.

[11] He H, Kiguchi K (2007). A study on emg-based control of exoskeleton robots for human lower-limb motion assist. 2007 6th International Special Topic Conference on Information Technology Applications in Biomedicine.

[12] Kawamoto H, Taal S, Niniss H, Hayashi T, Kamibayashi K, Eguchi K, Sankai Y (2010). Voluntary motion support control of robot suit hal triggered by bioelectrical signal for hemiplegia. 2010 Annual International Conference of the IEEE Engineering in Medicine and Biology.

[13] van Asseldonk EHF, van der Kooij H. Robot-Aided Gait Training with LOPES In: In: Dietz V, Nef T., Rymer WZ, editors. Londen: Springer: 2012. p. 379–96.

[14] Kazerooni H, Steger R, Huang L (2006). Hybrid control of the berkeley lower extremity exoskeleton (bleex). Int J Robot Res.

[15] Ajayi MO, Djouani K, Hamam Y (2014). Shank-foot trajectory control: A forward dynamics approach using computed-torque control. IEEE-RAS International Conference on Humanoid Robots.

[16] Vallery H, Veneman J, van Asseldonk E, Ekkelenkamp R, Buss M, van Der Kooij H (2008). Compliant actuation of rehabilitation robots. IEEE Robot & Autom Mag.

[17] Pratt GA, Williamson MM (1995). Series elastic actuators. Proceedings 1995 IEEE/RSJ International Conference on Intelligent Robots and Systems.

[18] Pratt GA, Willisson P, Bolton C, Hofman A (2004). Late motor processing in low-impedance robots: impedance control of series-elastic actuators. Proceedings of the 2004 American Control Conference.

[19] Mathijssen G, Cherelle P, Lefeber D, Vanderborght B (2013). Concept of a series-parallel elastic actuator for a powered transtibial prosthesis. Actuators.

[20] Quinlivan B. T., Lee S., Malcolm P., Rossi D. M., Grimmer M., Siviy C., Karavas N., Wagner D., Asbeck A., Galiana I., Walsh C. J. (2017). Assistance magnitude versus metabolic cost reductions for a tethered multiarticular soft exosuit. Science Robotics.

[21] Zhang J, Fiers P, Witte KA, Jackson RW, Poggensee KL, Atkeson CG, Collins SH (2017). Human-in-the-loop optimization of exoskeleton assistance during walking. Science.

[22] Collins SH, Wiggin MB, Sawicki GS (2015). Reducing the energy cost of human walking using an unpowered exoskeleton. Nature.

[23] Feng S, Whitman E, Xinjilefu X, Atkeson CG (2014). Optimization Based Full Body Control for the ATLAS Robot. 2014 IEEE-RAS International Conference on Humanoid Robots.

[24] Herzog A, Righetti L, Grimminger F, Pastor P, Schaal S (2014). Balancing experiments on a torque-controlled humanoid with hierarchical inverse dynamics. 2014 IEEE/RSJ International Conference on Intelligent Robots and Systems.

[25] Sherikov A, Dimitrov D, Wieber P. -B. (2014). Whole Body Motion Controller with Long-Term Balance Constraints. 14th IEEE-RAS International Conference on Humanoid Robots (Humanoids).

[26] Ott C, Roa MA, Hirzinger G (2011). Posture and balance control for biped robots based on contact force optimization. 11th IEEE-RAS International Conference on Humanoid Robots.

[27] Righetti L, Buchli J, Mistry M, Kalakrishnan M, Schaal S (2013). Optimal distribution of contact forces with inverse-dynamics control. Int J Robot Res.

[28] Carmichael MG, Liu D (2013). Estimating physical assistance need using a musculoskeletal model. IEEE Trans Biomed Engineer.

[29] Tucker MR, Olivier J, Pagel A, Bleuler H, Bouri M, Lambercy O, Millán JdR, Riener R, Gassert R (2015). Control strategies for active lower extremity prosthetics and orthotics: a review. J NeuroEngineering Rehabil.

[30] Afschrift Maarten, De Groote Friedl, De Schutter Joris, Jonkers Ilse (2014). The effect of muscle weakness on the capability gap during gross motor function: a simulation study supporting design criteria for exoskeletons of the lower limb. BioMedical Engineering OnLine.

[31] Unluhisarcikli O, Pietrusinski M, Weinberg B, Bonato P, Mavroidis C (2011). Design and control of a robotic lower extremity exoskeleton for gait rehabilitation. 2011 IEEE/RSJ International Conference on Intelligent Robots and Systems.

[32] Featherstone R. Rigid Body Dynamics Algorithms In: In: Featherstone R, editor. 1st. New York: Springer: 2008. p. 171–93.

[33] Spong MW (1987). Modeling and control of elastic joint robots. Trans ASME J Dynam Syst Meas Control.

[34] Swevers J, Ganseman C, Tükel DB, De Schutter J, Van Brussel H (1997). Optimal robot excitation and identification. IEEE Trans Robot Autom.

[35] Vantilt J, Aertbeliën E, De Groote F, De Schutter J (2015). Optimal excitation and identification of the dynamic model of robotic systems with compliant actuators. 2015 IEEE International Conference on Robotics and Automation.

[36] Palli G, Melchiorri C, Luca AD (2008). On the feedback linearization of robots with variable joint stiffness. 2008 IEEE International Conference on Robotics and Automation.

[37] Chung W, Fu L-C, Hsu S-H, In: Siciliano B, Khatib O. (2008). Motion control. Springer Handbook of Robotics.

[38] An CH, Atkeson CG, Griffiths JD, Hollerbach JM (1989). Experimental evaluation of feedforward and computed torque control. IEEE Trans Robot Autom.

[39] Ronsse R, Rossi SMMD, Vitiello N, Lenzi T, Carrozza MC, Ijspeert AJ (2013). Real-time estimate of velocity and acceleration of quasi-periodic signals using adaptive oscillators. IEEE Trans Robot.

[40] Tanghe K, Aertbeliën E, Vantilt J, Moltedo M, Bacek T, De Schutter J (2018). Realtime delayless estimation of derivatives of noisy sensor signals for quasi-cyclic motions with application to joint acceleration estimation on an exoskeleton. IEEE Robotics and Automation Letters (RA-L)(accepted).

[41] Wyeth G. Australasian Conference on Robotics and Automation In: In: MacDonald B, editor. Auckland: Australian Robotics and Automation Association Inc: 2006.

[42] der Kooij H. van, Veneman J.F., Ekkelenkamp R. (2006). Compliant Actuation of Exoskeletons. Mobile Robots: towards New Applications.

[43] Vallery H, Ekkelenkamp R, van der Kooij H, Buss M (2007). Passive and accurate torque control of series elastic actuators. Proceedings of the 2007 IEEE/RSJ International Conference on Intelligent Robots and Systems.

[44] Vantilt J, Giraddi C, Aertbeliën E, De Groote F, De Schutter J (2018). Estimating contact forces and moments for walking robots and exoskeletons using complementary energy methods. IEEE Robot Autom Lett.

[45] Paine N, Oh S, Sentis L (2014). Design and control considerations for high-performance series elastic actuators. IEEE/ASME Trans Mechatron.

[46] Junius K, Brackx B, Grosu V, Cuypers H, Geeroms J, Moltedo M, Vanderborght B, Lefeber D (2014). Mechatronic design of a sit-to-stance exoskeleton. Proceedings of the 5th IEEE RAS/EMBS International Conference on Biomedical Robotics and Biomechatronics.

[47] Roebroeck ME, Doorenbosch CAM, Harlaar J, Jacobs R, Lankhorst GJ (1994). Biomechanics and muscular activity during sit-to-stand transfer. Clinic Biomech.

[48] Brackx B (2014). Design of a modular add-on compliant actuator to convert an orthosis into an assistive exoskeleton. Proceedings of the 5th IEEE RAS/EMBS International Conference on Biomedical Robotics and Biomechatronics.

[49] EtherCAT Technology Group. EtherCAT. https://www.ethercat.org/default.htm.

[50] Xsens Technologies BV. Xsens. https://www.xsens.com.

[51] Tsukahara A, Kawanishi R, Hasegawa Y, Sankai Y (2010). Sit-to-stand and stand-to-sit transfer support for complete paraplegic patients with robot suit hal. Adv Robot.

[52] Mori Y, Okada J, Takayama K (2006). Development of a standing style transfer system “able” for disabled lower limbs. IEEE/ASME Trans Mechatron.

[53] Nakamura T, Saito K, Wang Z, Kosuge K (2005). Realizing model-based wearable antigravity muscles support with dynamics terms. 2005 IEEE/RSJ International Conference on Intelligent Robots and Systems.

[54] Tanghe K, Harutyunyan A, Aertbelien E, De Groote F, De Schutter J, Vrancx P, Nowe A (2016). Predicting seat-off and detecting start-of- assistance events for assisting sit-to-stand with an exoskeleton. IEEE Robot Autom Lett.

[55] Delp SL, Anderson FC, Arnold AS, Loan P, Habib A, John CT, Guendelman E, Thelen DG (2007). Opensim: Open-source software to create and analyze dynamic simulations of movement. IEEE Trans Biomed Engineer.

[56] Hermens HJ, Freriks B, Disselhorst-Klug C, Rau G (2000). Development of recommendations for semg sensors and sensor placement procedures. J Electromyography Kinesiol.

[57] Wilcoxon F (1945). Individual comparisons by ranking methods. Biomet Bullet.

[58] Holm S (1979). A simple sequentially rejective multiple test procedure. Scandinavian J Stat.

[59] Pataky PC, Robinson MA, Vanrenterghem J (2013). Vector field statistical analysis of kinematic and force trajectories. J Biomech.

